# GM-CSF-activated human dendritic cells promote type 1 T follicular helper cell polarization in a CD40-dependent manner

**DOI:** 10.1242/jcs.260298

**Published:** 2022-11-11

**Authors:** Sarantis Korniotis, Melissa Saichi, Coline Trichot, Caroline Hoffmann, Elise Amblard, Annick Viguier, Sophie Grondin, Floriane Noel, Hamid Mattoo, Vassili Soumelis

**Affiliations:** ^1^ Université de Paris, Inserm U976 HIPI Unit, Institut de Recherche Saint-Louis, F-75010 Paris, France; ^2^ Immunology and Inflammation Therapeutic Area, Sanofi, 94400 Vitry-sur-Seine, France; ^3^ Institut Curie, PSL University, 26 rue d'Ulm, F-75005 Paris, France; ^4^ Inserm U932 Research Unit Immunity and Cancer, Institut Curie, 26 rue d'Ulm, F-75005 Paris, France; ^5^ Immunology and Inflammation Therapeutic Area, Sanofi, Cambridge, MA 02142, USA; ^6^ Assistance Publique-Hôpitaux de Paris, Hôpital Saint-Louis, Laboratoire d'Immunologie, F-75010 Paris, France

**Keywords:** DC diversification, Human Tfh polarization, Primary blood conventional DC

## Abstract

T follicular helper (Tfh) cells regulate humoral responses and present a marked phenotypic and functional diversity. Type 1 Tfh (Tfh1) cells were recently identified and associated with disease severity in infection and autoimmune diseases. The cellular and molecular requirements to induce human Tfh1 differentiation are not known. Here, using single-cell RNA sequencing (scRNAseq) and protein validation, we report that human blood CD1c^+^ dendritic cells (DCs) activated by GM-CSF (also known as CSF2) drive the differentiation of naive CD4^+^ T cells into Tfh1 cells. These Tfh1 cells displayed typical Tfh molecular features, including high levels of PD-1 (encoded by *PDCD1*), CXCR5 and ICOS. They co-expressed BCL6 and TBET (encoded by *TBX21*), and secreted large amounts of IL-21 and IFN-γ (encoded by *IFNG*). Mechanistically, GM-CSF triggered the emergence of two DC sub-populations defined by their expression of CD40 and ICOS ligand (ICOS-L), presenting distinct phenotypes, morphologies, transcriptomic signatures and functions. CD40^High^ ICOS-L^Low^ DCs efficiently induced Tfh1 differentiation in a CD40-dependent manner. In patients with mild COVID-19 or latent *Mycobacterium tuberculosis* infection, Tfh1 cells were positively correlated with a CD40^High^ ICOS-L^Low^ DC signature in scRNAseq of peripheral blood mononuclear cells or blood transcriptomics, respectively. Our study uncovered a novel CD40-dependent Tfh1 axis with potential physiopathological relevance to infection.

This article has an associated First Person interview with the first author of the paper.

## Teaser

**Summary:** A newly described mechanism for human Tfh1 polarization with possible implications in infection.

## INTRODUCTION

T follicular helper (Tfh) cells provide critical help to B cells for proliferation, somatic hypermutation, class-switch recombination and differentiation into antibody-producing plasma cells (Schwickert et al., 2011; Yeh et al., 2018). They have been associated with several human diseases, including viral and bacterial infections (Fousteri and Kuka, 2020; Rodrigues et al., 2014; Juno et al., 2020; Gong et al., 2020; Zhang et al., 2020b; Koutsakos et al., 2018), asthma (Gong et al., 2019; Gowthaman et al., 2019; Ma et al., 2017), cancer (Gu-Trantien et al., 2017; Byford et al., 2018; Meng et al., 2018) and autoimmune diseases (Schafflick et al., 2020; Seth and Craft, 2019; Gensous et al., 2018), but the mechanisms underpinning their development and functions are not well characterized.

Circulating Tfh cells can be divided into sub-populations that share key phenotypic and functional characteristics with other T helper (Th) cell lineages – such as Th1, Th2 and Th17 – and that display different capacities in regulating B cell responses (Morita et al., 2011). The nature of the inflammatory microenvironment affects Tfh differentiation programs, which may subsequently regulate B cell immunity. Type 1 Tfh (Tfh1) cells were identified based on their cytokine profile, which is characterized by the co-production of IL-21 and IFN-γ (encoded by *IFNG*), and specific phenotypic features, namely expression of PD-1 (encoded by *PDCD1*), ICOS, CXCR5 and CXCR3, and absence of CCR6 (Morita et al., 2011). Numbers of Tfh1 cells are increased in HIV infection, where the Tfh1 population represents a major fraction of the viral reservoir (Baiyegunhi et al., 2018; Martin-Gayo et al., 2020; Velu et al., 2018). Studies of *Mycobacterium tuberculosis* (MTB) infection have revealed a role of Tfh1 cells and an association with disease severity (Jasenosky et al., 2015; Li et al., 2016; Kumar et al., 2014; Slight et al., 2013). Mouse studies have shown an important role of Tfh1 cells in controlling Zika virus infection, as well as lymphocytic choriomeningitis virus (LCMV) infection, mainly through IFN-γ secretion (Weinstein et al., 2018; Liang et al., 2019). Additionally, it has been recently shown that circulating Tfh1 cells are positively correlated with the magnitude of viral-specific antibodies in both influenza and COVID-19 patients (Juno et al., 2020; Gong et al., 2020; Zhang et al., 2020a,b; Martin-Gayo et al., 2017; Bentebibel et al., 2013). In line with results from studies of influenza vaccination, recently published findings in mice have revealed that immunization with SARS-CoV-2 mRNA elicits potent viral-specific Tfh1 cells needed to produce long-lived plasma cells (Bentebibel et al., 2013; Lederer et al., 2020; Laczkó et al., 2020).

Tfh differentiation requires cooperation between antigen-specific interactions and signaling pathways, co-stimulation, cytokines and chemokine receptors (Crotty, 2014). Dendritic cell (DC)-derived cytokines, such as IL-6, IL-12, IL-23 and TGF-β, promote surface CXCR5 expression on Tfh cells, facilitating their migration towards the T cell–B cell interface within the secondary lymphoid organ germinal centers (GCs) (Papillion et al., 2019; Crotty, 2011). Additionally, human Tfh differentiation is driven by activin-A in a SMAD2- and SMAD3-dependent manner (Locci et al., 2016). Given the established Tfh subset diversity, it is now of critical physiopathological and therapeutic importance to identify cellular and molecular mechanisms controlling specific Tfh differentiation pathways. We have previously shown that TSLP-activated DCs can promote type 2 Tfh (Tfh2) cell differentiation through OX40 ligand (also known as TNFSF4; Pattarini et al., 2017). However, the factors inducing human Tfh1 differentiation remain elusive.

Here, single-cell RNA sequencing (scRNAseq) analysis of human CD4^+^ T cells differentiated by GM-CSF (CSF2)-activated blood DCs (GM-CSF-DCs) revealed the presence of bona fide Tfh1 cells. Mechanistically, GM-CSF induced diversification of human DCs into two phenotypically, transcriptionally and functionally distinct subsets. Only DCs with high levels of CD40 and low levels of ICOS ligand (ICOS-L) could efficiently drive Tfh1 polarization, which occurred in a CD40-dependent manner. Moreover, we found that Tfh1 cells were positively correlated with a signature of CD40^High^ ICOS-L^Low^ DCs in two different clinical settings of infection: patients with MTB infection and active COVID-19 patients. Overall, our results define a novel Tfh1 differentiation pathway, as well as potential molecular targets for its pharmacological manipulation.

## RESULTS

### scRNAseq reveals a Tfh1 polarization program induced by GM-CSF-activated DCs

**Fig. 1.**
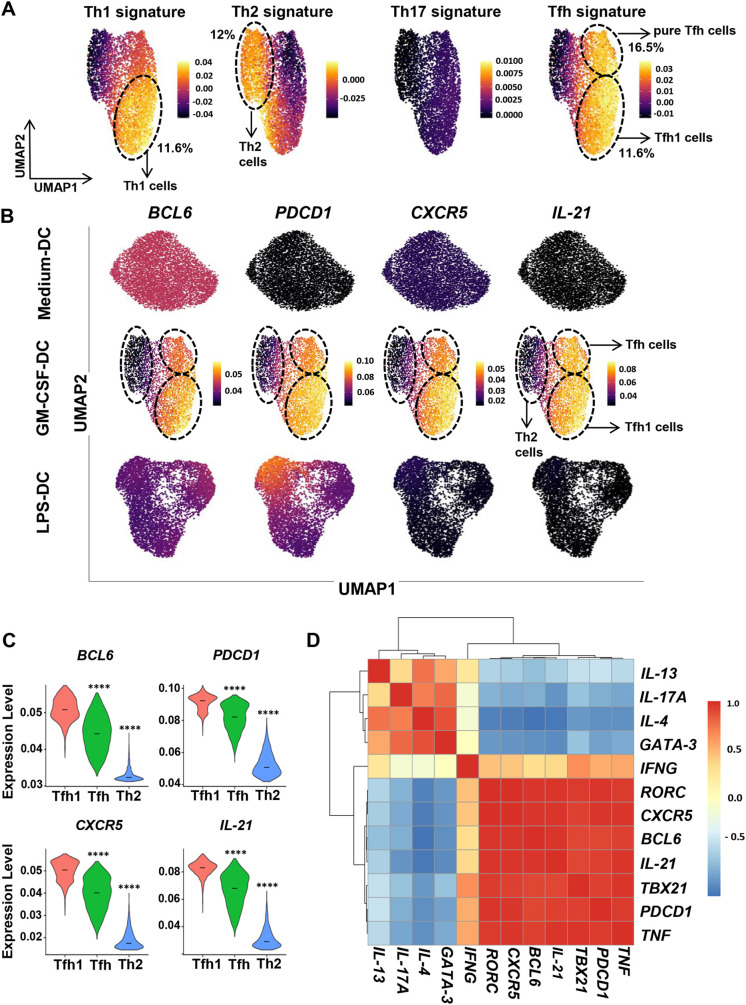
**scRNAseq reveals a Tfh1 polarization program induced by GM-CSF-activated DCs.** (A) UMAP plots color-coded to show the expression scores of Th1, Th2, Th17 and Tfh signatures (Table S1) in CD4^+^ T cells differentiated by co-culture with GM-CSF-DCs. The percentage of cells in each of the indicated subpopulations (dashed ovals) are shown on the figure. (B) UMAP plots of scRNAseq of CD4^+^ T cells differentiated *in vitro* by co-culture with Medium-DCs, GM-CSF-DCs or LPS-DCs. For each plot, the cells are color-coded according to their expression levels of the indicated Tfh-related genes. (C) Violin plot representation of the expression level of *BCL6*, *PDCD1* (which encodes PD-1), *CXCR5* and *IL-21* in the Tfh-, Tfh1- and Th2-enriched clusters. Horizontal bars indicate the mean. ****P<0.0001 compared with Tfh1. (D) Pearson correlation matrix of the expression values of the indicated Tfh-, Th1-, Th2- and Th17-related genes within CD4^+^ T cells differentiated by co-culture with GM-CSF-DCs.

We decided to revisit human Th cell differentiation induced by GM-CSF-DCs in a comprehensive manner using scRNAseq. Primary human naive CD4^+^ T lymphocytes were co-cultured for 6 days with allogeneic primary blood type 2 conventional DCs (cDC2) that had been either activated for 24 h with lipopolysaccharide (LPS; resulting in LPS-DCs) or GM-CSF (resulting in GM-CSF-DCs), or cultured only in medium (resulting in Medium-DCs). scRNAseq was performed in sorted activated T cells after 6 days of co-culture, using the 10X Genomics platform, on an average of 5600 cells per DC condition. This led to a total of 17,070 high-quality sequenced cells, with an average of 4000 detected genes per cell. To probe the dataset for prototypical Th subsets, we used knowledge-driven signatures characteristic of Th1, Th2, Th17 and Tfh cells (Table S1). To dissect Th diversity, we applied these signatures to CD4^+^ T cells generated with GM-CSF-DCs. We reduced the dimensionality of the data using principal component analysis (PCA) then used uniform manifold approximation and projection (UMAP) to visualize the data. Th1 and Th2 signatures were enriched in distinct cell clusters, representing 11.6% and 12.0% of cells, respectively (Fig. 1A). The Th17 signature was not enriched in the whole dataset, indicating that GM-CSF-DCs do not have the potential to induce the Th17 differentiation program. Interestingly, two clusters were enriched in the Tfh signature. One of them co-expressed a Th1 signature, suggestive of Tfh1 cells (11.6% of cells), while the other (16.5% of cells) did not overlap with the Th1- or Th2-enriched clusters (Fig. 1A). Tfh-enriched clusters were not detected in T cells activated by either Medium-DCs or LPS-DCs (Fig. S1A). To better define the specific contribution of Tfh markers to the Tfh signature enrichment in the different cell clusters, we represented the expression of four key Tfh marker genes: *BCL6*, *PDCD1*, *CXCR5* and *IL21*. All four genes were highly expressed exclusively in CD4^+^ T cells differentiated by GM-CSF-DCs (Fig. 1B; *P*=0; permutation test comparing GM-CSF-DC and LPS-DC conditions, and GM-CSF-DC and Medium-DC conditions). Interestingly, the Tfh1-enriched cluster displayed higher expression of genes encoding both the phenotypic markers PD-1 and CXCR5, the transcription factor BCL6 and the key cytokine IL-21, suggesting the induction of a stronger Tfh polarization within Tfh1 cells (Fig. 1C). The induction of Tfh1 cells by GM-CSF-DCs was additionally validated by using Pearson pairwise correlation matrices for phenotypic markers, transcription factors and cytokines known to characterize the three major Th cell lineages Th1, Th2 and Th17. We observed a high correlation between expression of the cytokines and the transcription factors, which is typical of Th1 and Tfh cells, within the *in vitro*-generated Tfh cells. *IL21* expression was strongly associated with expression of *IFNG* and *TNFA* (also known as *TNF*), which encode Th1-related cytokines, but not with expression of *IL4* or *IL17A*, which encode Th2- and Th17-related cytokines, respectively. Similarly, expression of *BCL6*, which encodes the master transcription factor of Tfh cells, was highly associated with expression of *TBX21*, which encodes the TBET transcription factor of Th1 cells, but not with expression of *GATA3*, a transcription factor of Th2 cells. Additionally, expression levels of the classical phenotypic markers of Tfh cells, *PDCD1* and *CXCR5*, displayed high correlation with levels of *IL21*, *IFNG*, *TNFA*, *BCL6* and *TBX21*, but not of *IL4*, *IL13*, *IL17A* and *GATA3* (Fig. 1D). When the expression level of these genes was represented separately, we observed that *GATA3* and *IL4* were exclusively expressed in the Th2-enriched cluster without major expression of *IL13* (Fig. S1B,C). *TNFA*, *IFNG* and *TBX21* displayed higher levels within the Tfh1-enriched cluster, which highlighted the importance of their co-expression in promoting efficient Tfh1 differentiation (Fig. S1B,C). Overall, our scRNAseq analysis revealed that GM-CSF-DCs polarized a significant fraction of naive CD4^+^ T cells into Tfh1 cells at the transcriptomic level.

### GM-CSF-DC-activated CD4^+^ T cells display a Tfh1 cytokine profile

**Fig. 2.**
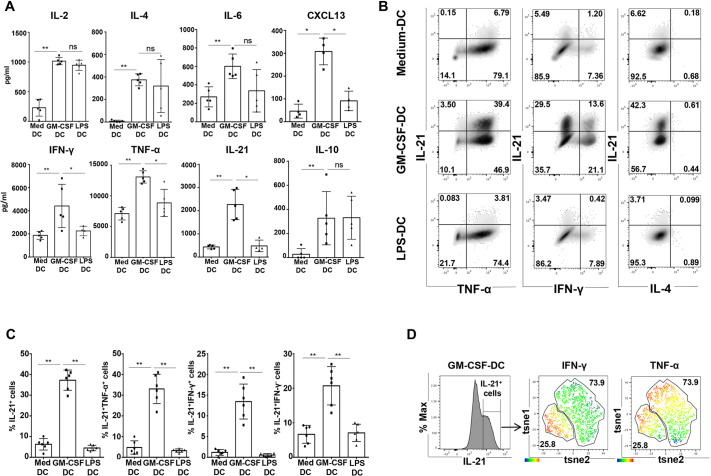
**GM-CSF-DC-activated CD4^+^ T cells display a Tfh1 cytokine profile.** (A) T cell cytokine quantification (using CBA, except IL-21 and CXCL13, which were quantified using ELISAs) after co-culture of naive CD4^+^ T cells with Medium-DCs (Med DC), GM-CSF-DCs or LPS-DCs. Mean±s.e.m. from *n*=5. (B) Intracellular FACS staining for T cell cytokines after co-culture with the indicated DCs. Plots represent cells gated in CD4^+^ live cells for one representative donor. The percentage of cells in each gate is indicated on the plots. (C) Percentages of IL-21^+^, IL-21^+^ TNF-α^+^, IL-21^+^ IFN-γ^+^, and IL-21^−^ IFN-γ^−^ cells as shown in B. Mean±s.e.m. from *n*=6. (D) IL-21 production in CD4^+^ T cells measured by FACS (left) and t-SNE analysis of IL-21^+^ cells, identifying two clusters with different expression levels of IFN-γ (middle) and TNF-α (right) for one representative donor. *P<0.05; **P<0.01; ns, not significant (two-tailed paired Student's *t*-test).

To validate our findings at the protein level, we characterized the secretion pattern of the GM-CSF-DC-induced T cell population. To do so, we differentially stimulated DCs for 24 h, and then we co-cultured them with allogeneic naive CD4^+^ T cells, as for the previous scRNAseq experiment. After 6 days of co-culture, T cells were re-stimulated with anti-CD3 and anti-CD28 (aCD3/aCD28)-coated beads for 24 h to measure cytokine secretion in the supernatants, or with phorbol myristate acetate (PMA), ionomycin and brefeldin A for 4 h before permeabilization for intracellular cytokine staining (ICS) and analysis by flow cytometry (Fig. S2A). We found that T cells co-cultured with GM-CSF-DCs secreted high levels of CXCL13, IFN-γ, TNF-α (also known as TNF) and IL-21, as compared to levels secreted by T cells co-cultured with either LPS-DCs or Medium-DCs (Fig. 2A). The high secretion of IL-21 and CXCL13 confirmed the induction of Tfh-like cells. The concomitant secretion of IFN-γ and TNF-α suggested the possibility of a Tfh1 polarization. GM-CSF-DC-activated T cells produced more IL-9 than LPS-DC-activated T cells, with no significant difference in the secretion of IL-4, IL-10 and IL-13, which are prototypical Th2 cytokines, and of IL-17A and IL-17F, which characterize the Th17 lineage. (Fig. 2A; Fig. S2B). These data supported the hypothesis that Tfh1 were being induced by GM-CSF-DCs, although at this stage we could not exclude the co-existence of Tfh and Th1 cells within the same T cell population. To investigate this, we studied T cell cytokine co-production at the single-cell protein level using ICS. We observed that only GM-CSF-DCs induced a high proportion of IL-21-producing T cells (37.2%±1.99; mean±s.e.m.) co-producing high levels of IFN-γ (13.46%±1.72) and TNF-α (33.04%±2.89) (Fig. 2B,C). We could not identify any cells co-producing IL-21 with either IL-4 or IL-17A, confirming our hypothesis that GM-CSF-DCs favored differentiation towards a Tfh1 fate (Fig. 2B,C; Fig. S2C). Our results indicated that GM-CSF-DCs were also inducers of IFN-γ^+^ IL-21^−^ Th1 cells (20.72%±2.27) (Fig. 2B,C). Unsupervised t-SNE analysis of IL-21-producing T cells revealed two clusters: the IFN-γ^+^ population (25.8%), which represents Tfh1 cells, and the IFN-γ^−^ population (73.9%), which represents pure Tfh cells (Fig. 2D). This analysis also revealed that both IL-21^+^ IFN-γ^+^ and IL-21^+^ IFN-γ^−^ cells produced TNF-α to a similar extent. These results validated our scRNAseq finding on the induction of Tfh1 cells by GM-CSF-DCs.

### GM-CSF-DC-induced T cells have phenotypic and functional features of Tfh1 cells

**Fig. 3.**
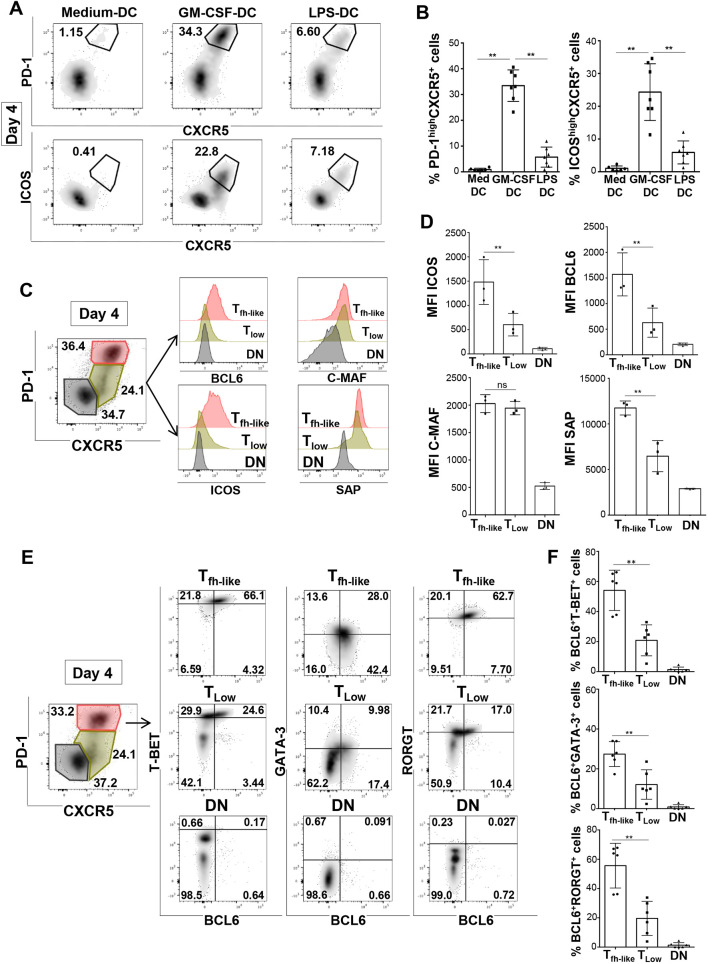
**GM-CSF-DC-induced T cells have phenotypic and functional features of Tfh1 cells.** (A) PD-1, ICOS and CXCR5 FACS analysis in CD4^+^ T cells at day 4 of co-culture with Medium-DCs, GM-CSF-DCs or LPS-DCs for one representative donor. Gates indicate PD-1^High^ CXCR5^+^ cells (top row) and ICOS^High^ CXCR5^+^ cells (bottom row), with percentage of cells within the gate shown. (B) Percentages of PD-1^High^ CXCR5^+^ and ICOS^High^ CXCR5^+^ cells as shown in A. Med DC, co-cuture with Medium-DCs. Mean±s.e.m. from *n*=7. (C) Identification of three populations in T cells differentiated by co-culture with GM-CSF-DCs for 4 days. T_fh-like_ cells (PD-1^High^ CXCR5^+^) are shown in red, T_Low_ cells (PD-1^Low^ CXCR5^+^) are shown in green and double-negative cells (DN; PD-1^−^ CXCR5^−^) are shown in black. FACS histograms show the expression of BCL6, C-MAF, ICOS and SAP in each of the three populations for one representative experiment. (D) BCL6, C-MAF, ICOS and SAP MFI quantification as shown in C. Mean±s.e.m. from *n*=3. (E) Intranuclear staining for the expression of BCL6, TBET, GATA3 and RORγT (RORGT) in T_fh-like_, T_Low_ (PD-1^Low^) and DN cells (color-coded as in C) induced by GM-CSF-DCs for one representative donor. The percentage of cells within each gate is shown on the plots. (F) Percentages of BCL6^+^ TBET^+^, BCL6^+^ GATA3^+^ and BCL6^+^ RORγT^+^ cells from data as shown in E. Mean±s.e.m. from *n*=6. **P<0.01; ns, not significant (two-tailed paired Student's *t*-test).

Next, we investigated the phenotype of GM-CSF-DC-activated T cells at the optimal time point of day 4 of co-culture (Pattarini et al., 2017). Among total activated CD4^+^ T cells in the GM-CSF-DC condition, we detected a very significant population of differentiated T cells with a phenotype of Tfh cells, co-expressing PD-1 with CXCR5 (38.58%±3.84; mean±s.e.m.) and ICOS with CXCR5 (29.175%±3.08) (Fig. 3A,B). Conversely, we observed that both non-activated DCs (Medium-DC) and LPS-DCs were much less efficient in inducing the expression of those three Tfh markers (Fig. 3A,B). At this time point, we detected three different activated T cell populations based on the expression levels of PD-1 and CXCR5: (1) T_fh-like_ cells, identified as PD-1^High^ CXCR5^+^; (2) T_Low_ (PD-1^Low^) cells, characterized by a PD-1^Low^ CXCR5^+^ phenotype; and (3) a PD-1^−^ CXCR5^−^ double-negative population (DN) (Fig. 3C). We examined the expression levels of other Tfh markers in these three populations. As expected, BCL6, a transcription factor needed for the development of Tfh cells, was highly expressed exclusively in T_fh-like_ cells. T_fh-like_ cells were also positive for ICOS, SAP (also known as SH2D1A) and C-MAF (also known as MAF), three additional factors used for the identification of functional Tfh cells (Ji et al., 2020) (Fig. 3C,D). We then asked the question whether these T_fh-like_ cells exclusively expressed their master transcription factor BCL6 or whether they expressed additional transcription factors of other Th cell lineages – TBET (Th1 lineage), RΟRγT (isoform 2 of RORγ, encoded by *RORC*; Th17 lineage) and GATA3 (Th2 lineage) – at the protein level. We observed that a very significant percentage of T_fh-like_ cells co-expressed BCL6 with either TBET (54.08%±5.47; mean±s.e.m.) or RΟRγt (55.61%±6.23) (Fig. 3E,F). The expression of RΟRγt might only be a result of transient activation of T cells, since the expression of this transcription factor was not associated with significant secretion of Th17-related cytokines, such as IL-17A and IL-17F, in supernatants or intracellularly (Fig. S2B,C). The percentage of cells co-expressing BCL6 and GATA3 was low (27.40%±2.56), fitting well with the absence of IL-4 secretion. Conversely, in both T_Low_ (PD-1^Low^) and DN cells, the co-expression of BCL6 with any of these three transcription factors was significantly lower, confirming that the *in vitro*-induced PD-1^High^ CXCR5^+^ T_fh-like_ cells were the only population displaying phenotypic features of Tfh cells (Fig. 3E,F). Next, we wanted to verify that the production of Tfh1-related cytokines (IFN-γ and IL-21) derived exclusively from the T_fh-like_ population. Sorted T_fh-like_ and T_Low_ (PD-1^Low^) cells were stimulated with PMA, ionomycin and brefeldin A for 4 h and stained intracellularly for IL-21, TNF-α, IFN-γ, TBET and BCL6. As expected, only T_fh-like_ cells co-produced IL-21 and TNF-α (Fig. S2D,E). Among them, 19.60%±3.80 (mean±s.e.m.) of cells expressed IFN-γ. Those cells were also positive for both BCL6 and TBET, confirming the Tfh1 polarization. Functionally, we asked whether the GM-CSF-DC-generated T_fh-like_ cells were able to induce the differentiation of B cells into plasma cells. Sorted T_fh-like_ and T_Low_ (PD-1^Low^) cells were co-cultured with either allogeneic naive B cells or allogenic memory B cells (Fig. S3A). After 10 days of co-culture, we identified significant proportions of CD19^Low^ CD38^High^ CD27^High^ cells, which represent plasma cells, only in the T_fh-like_ co-culture condition. More specifically, we found that T_fh-like_ cells induced differentiation of memory B cells into plasma cells. The extent of this plasma cell differentiation was comparable to the one obtained with CpG-B-activated memory B cells, clearly showing the ability of T_fh-like_ cells to exert prototypical Tfh functions. On the other hand, T_Low_ (PD-1^Low^) cells promoted very low levels of plasma cell differentiation with both naive and memory B cells (Fig. S3A,B). These findings confirm that GM-CSF-DC co-culture is a new experimental condition allowing the induction of both phenotypical and functional T_fh-like_ cells.

### GM-CSF induces two DC populations with different phenotypes, morphologies and plasticities

**Fig. 4.**
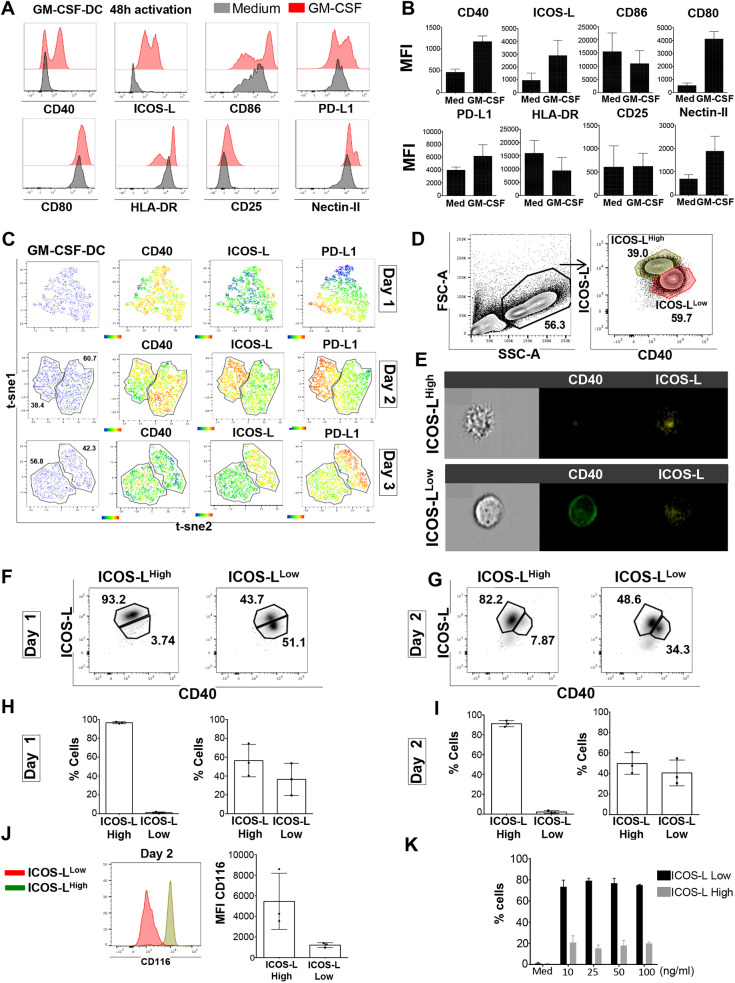
**GM-CSF induces the emergence of two populations of DCswith different phenotypes, morphologiesand plasticities.** (A) Phenotypic FACS analysis of GM-CSF-DCs and Medium-DCs for one representative donor at day 2 post *in vitro* stimulation. (B) MFI quantification for data as shown in A (mean±s.e.m., *n*=6). Med, stimulation with medium only. (C) T-SNE analysis of GM-CSF-DCs for the identification of different cell clusters at different time points of stimulation (day 1, day 2 and day 3) based on the expression of CD40, ICOS-L and PD-L1 for one representative experiment. Cell clusters are outlined in the plots, with the percentage of cells in each cluster indicated. (D) FACS analysis for the detection and cell sorting of two different GM-CSF-induced DC sub-populations for one representative experiment. Cell sorting was used based exclusively on the expression of CD40 and ICOS-L. Percentage of cells in each sub-population is shown on the plot. (E) Imaging flow cytometry analysis of ICOS-L^High^ and ICOS-L^Low^ GM-CSF-DCs, displaying morphology and expression of CD40 and ICOS-L for one representative donor (*n*=3). (F,G) ICOS-L and CD40 expression by ICOS-L^High^ and ICOS-L^Low^ GM-CSF-DCs after (F) 1 day or (G) 2 days of GM-CSF re-stimulation for one representative experiment. The percentage of cells in each gate is indicated. (H,I) Percentages of ICOS-L^High^ and ICOS-L^Low^ cells as shown in F and G, respectively (mean±s.e.m., *n*=3). (J) Left: GM-CSF receptor α chain (CD116) expression in ICOS-L^High^ (green) and ICOS-L^Low^ (red) cells for one representative experiment. Right: bar graph showing the mean±s.e.m. CD116 MFI from four independent experiments. (K) Percentages of ICOS-L^High^ and ICOS-L^Low^ subsets of DCs recovered after 48 h of DC activation with different doses of GM-CSF (mean±s.e.m., *n*=5).

To explore further the mechanism used by GM-CSF-DCs to induce Tfh1 differentiation, we sought to characterize the maturation profile of GM-CSF-DCs. After 48 h of activation, GM-CSF induced strong upregulation of CD40, ICOS-L, CD86 and PD-L1 (also known as CD274) and intermediate upregulation of CD80, HLA-DR, CD25 (also known as IL2RA) and nectin-II (nectin2) as compared to levels in Medium-DCs (Fig. 4A,B). The presence of two peaks of expression for some of these markers over time suggested the existence of two DC sub-populations. T-SNE analysis of total GM-CSF-DCs at day 1, day 2 and day 3 showed that GM-CSF induced the emergence of two different activated sub-populations from day 2 (Fig. 4C). One population expressed high levels of CD40 and low levels of ICOS-L and PD-L1 (referred to hereafter as ICOS-L^Low^), whereas the second population expressed low levels of CD40 and high levels of ICOS-L and PD-L1 (referred to hereafter as ICOS-L^High^) (Fig. 4C). Interestingly, the percentage of DCs in the ICOS-L^Low^ population was higher than the percentage of DCs in the ICOS-L^High^ population at both day 2 and day 3 of GM-CSF stimulation (45.180%±4.14 at day 2 and 39.45%±4.0 at day 3 for ICOS-L^High^, 54.06%±4.0 at day 2 and 59.12%±2.2 at day 3 for ICOS-L^Low^; mean±s.e.m.; Fig. S4A). For further analysis, we sorted the two sub-populations at day 2 using CD40 and ICOS-L staining (Fig. 4D). Using a flow-stream imaging approach, we detected morphological differences between the two sub-populations. The ICOS-L^High^ DCs displayed a typical morphology of an activated DC, with high forward scatter (FSC) and side scatter (SSC) levels, typical dendrites and very low levels of circularity. The ICOS-L^Low^ DCs were rounder, with low FSC/SSC and no dendrites, suggesting a less mature stage of differentiation (Fig. 4E; Fig. S4B). To address the question of cell plasticity, sorted ICOS-L^High^ and ICOS-L^Low^ DCs at day 2 were re-stimulated with GM-CSF for a further 48 h. We observed a stable phenotype of ICOS-L^High^ DCs after both 24 h and 48 h of re-stimulation (Fig. 4F,G). Conversely, ICOS-L^Low^ DCs were more plastic, since 56.3%±9.9 (mean±s.e.m.) of cells at 24 h and 49.8%±6.0 of cells at 48 h acquired the ICOS-L^High^ DC phenotype (Fig. 4F–I). We then thought to analyze the expression of GM-CSF receptor α chain (CD116, also known as CSF2RA) in the two sub-populations of GM-CSF-DCs, as well as in Medium-DCs and LPS-DCs. ICOS-L^High^ DCs displayed higher levels of CD116 compared to those observed for ICOS-L^Low^ DCs, which might contribute to the lower secondary response of ICOS-L^Low^ DCs to GM-CSF exposure (Fig. 4J). We observed that total GM-CSF-DCs displayed two peaks of CD116 expression at both day 1 and day 2, suggesting that this marker could be also used to separate the two GM-CSF-induced sub-populations. ICOS-L^High^ (CD116^+^) DCs expressed lower levels of CD166 than the LPS-DCs but comparable levels to those observed for Medium-DCs, whereas the ICOS-L^Low^ DCs (CD116^Low^) downregulated the expression of CD166 (Fig. S4C). We hypothesized that the emergence of these two sub-populations could also be dose dependent. To address this hypothesis, we activated DCs for 48 h with increasing doses of GM-CSF (10–100 ng/ml). The emergence of the two sub-populations was independent of the GM-CSF dose, since the ratio between ICOS-L^High^ and ICOS-L^Low^ DCs (10:6) did not vary between the different doses tested (Fig. 4K). These data raised the question of whether the GM-CSF-DC-induced sub-populations also displayed functional differences in promoting Tfh polarization.

### GM-CSF-induced ICOS-L^Low^ DCs promote Tfh1 polarization

**Fig. 5.**
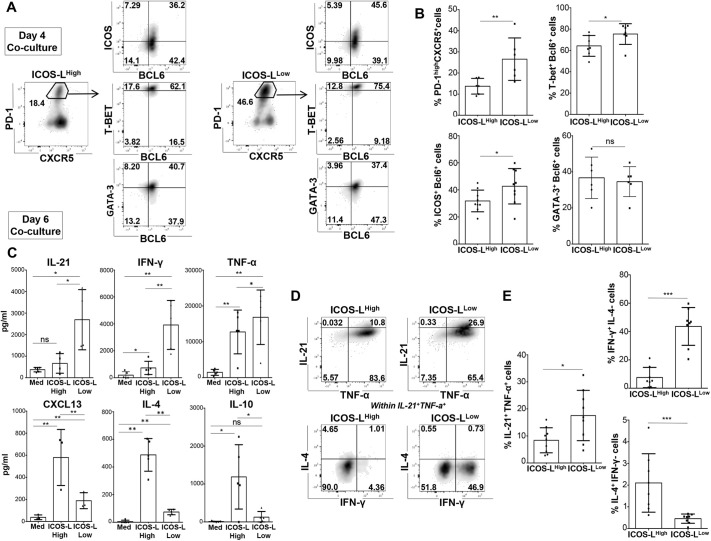
**CD40-dependent Tfh1 polarization by GM-CSF-DCs.** (A) BCL6, TBET, GATA3 and ICOS FACS analysis in PD-1^High^ CXCR5^+^ T cells activated either by ICOS-L^High^ (left) or ICOS-L^Low^ (right) GM-CSF-DCs for one representative experiment (day 4 of co-culture). The percentage of cells in each gate is indicated on the plots. (B) Percentages of PD-1^High^ CXCR5^+^ T cells and of ICOS^+^ BCL6^+^, TBET^+^ BCL6^+^ and GATA3^+^ BCL6^+^ cells gated in the PD-1^High^ CXCR5^+^ T cells, as shown in A. Mean±s.e.m. *n*=6. (C) T cell cytokine quantification after co-culture of naive CD4^+^ T cells with ICOS-L^High^ or ICOS-L^Low^ GM-CSF-DCs, or with Medium-DCs (Med) as a control. Mean±s.e.m. *n*=5. (D) Intracellular FACS staining for T cell cytokines. Plots represent cells gated in CD4^+^ live cells for one representative donor. Cell were activated either by ICOS-L^High^ (left) or ICOS-L^Low^ (right). Analysis for IL-4 and IFN-γ is gated in IL-21^+^ TNF-α^+^ cells for one representative donor. The percentage of cells in each gate is indicated on the plots. (E) Percentages of IL-21^+^ TNF-α^+^ cells and of IFN-γ^+^ IL-4^−^ and IFN-γ^−^ IL-4^+^ cells gated in IL-21^+^ TNF-α^+^ cells from data as shown in D. Mean±s.e.m. from *n*=8. *P<0.05; **P<0.01; ***P<0.001; ns, not significant (two-tailed paired Student's *t*-test).

We performed 4-day and 6-day co-cultures of sorted ICOS-L^High^ and ICOS-L^Low^ cells with allogeneic naive CD4^+^ T cells. At day 4, we observed that ICOS-L^Low^ DCs were more efficient in driving Tfh differentiation, since 29.6%±4.6 (mean±s.e.m.) of cells displayed a Tfh phenotype in the ICOS-L^Low^ DC condition compared to 13.5%±1.3 of cells in the ICOS-L^High^ DC condition (Fig. 5A,B). Within T_fh-like_ cells, ICOS-L^Low^ DCs induced higher co-expression of BCL6 with either TBET (75.4%±3.7; mean±s.e.m.) or ICOS (42.7%±4.3), as compared to expression in ICOS-L^High^ DC-activated T cells (64.28%±3.69 for BCL6 with TBET and 31.8%±2.7 for BCL6 with ICOS). GATA3 was also co-expressed with BCL6 but at lower levels (58.4%±3.2 for ICOS-L^Low^ and 61.9%±4.2 for ICOS-L^High^) (Fig. 5A,B). The higher percentage of PD-1^high^ CXCR5^+^ T_fh-like_ cells induced by ICOS-L^Low^ DCs together with a significant co-expression of BCL6, ICOS and TBET within this population, suggested that this subset displayed the strongest potential to induce a Tfh1 phenotypic profile. Next, we studied the cytokine production of T cells activated by each GM-CSF-DC sub-population at day 6, either in supernatants or by ICS. We observed that ICOS-L^Low^ DC-activated T cells secreted high amounts of IL-21, IFN-γ, TNF-α and IL-12p70, whereas ICOS-L^High^ DC-activated T cells secreted more CXCL13, IL-4, IL-10, IL-9 and IL-13 (Fig. 5C; Fig. S5). Additionally, ICS showed that ICOS-L^Low^ DCs induced higher co-secretion of IL-21 and TNF-α by T cells (17.5%±3.1; mean±s.e.m.) as compared to the ICOS-L^High^ DC condition (8.4%±1.5). Among IL-21-producing T cells, ICOS-L^Low^ DCs promoted a high production of IFN-γ (46.6%±4.7) but not IL-4 (0.4%±0.1). Conversely, ICOS-L^High^ DCs induced significant percentage of IL-4-secreting T_fh-like_ cells (2.0%±0.5) but much lower levels of IFN-γ production (7.5%±2.5) as compared to that induced by ICOS-L^Low^ DCs (Fig. 5D,E). Based on the cytokine profile of T cells, we concluded that ICOS-L^Low^ DCs were efficient inducers of Tfh polarization, favoring the differentiation into Tfh1, whereas ICOS-L^High^ DCs only promoted formation of a few Tfh and/or Th2 cells. There was no significant difference in the T cell polarization induced by total GM-CSF-DCs as compared to the one induced by ICOS-L^Low^ DCs suggesting that this sub-population dominated over the ICOS-L^High^ DCs. This can be explained either by the higher number of ICOS-L^Low^ DCs (Fig. S4A) or by a cytokine competition that favors the induction of a Tfh1 profile.

### RNA sequencing reveals major transcriptomic differences between ICOS-L^High^ and ICOS-L^Low^ DCs

**Fig. 6.**
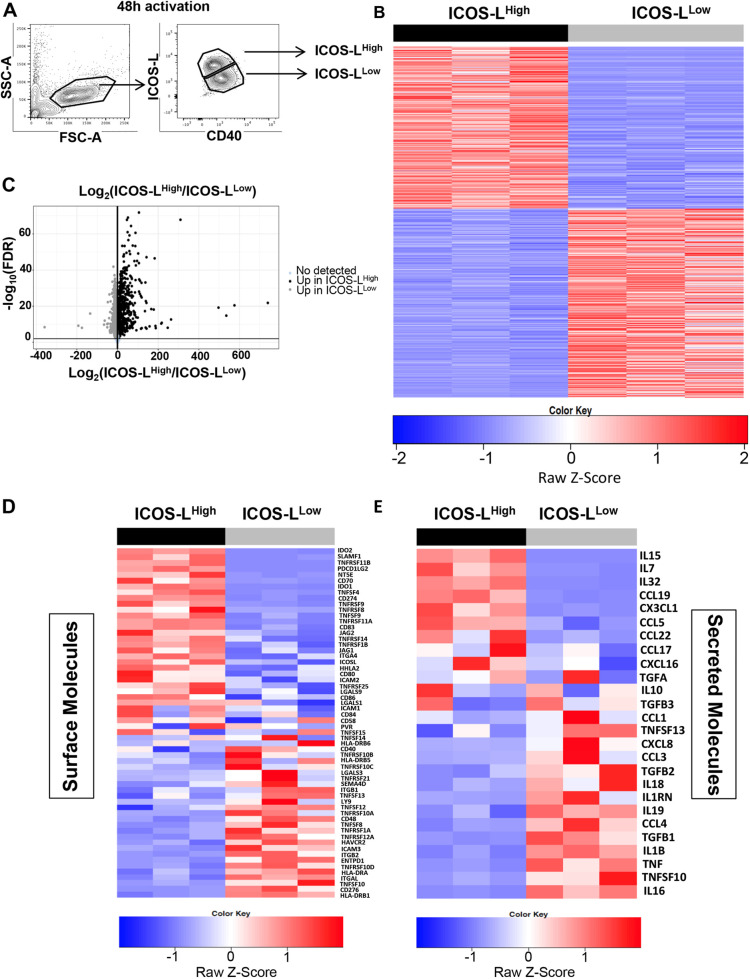
**RNAseq reveals major transcriptomic differences between GM-CSF-activated ICOS-L^High^ and ICOS-L^Low^ DCs.** (A) Representative plots showing cell sorting strategy for the collection of ∼100,000 ICOS-L^High^ and ICOS-L^Low^ DCs from three independent donors (*n*=3) at day 2 of GM-CSF stimulation. (B) Heatmap representation of DEGs between ICOS-L^High^ and ICOS-L^Low^ DCs from three donors, color-coded based on *Z*-score values, with high expression in red and low expression in blue. Each row represents a gene, and each column for ICOS-L^High^ and ICOS-L^Low^ represents a sample from an independent donor. (C) Volcano plot of DEGs represented through their respective log_2_ fold-change (*x*-axis) plotted against the false discovery rate (FDR; *y*-axis). Upregulated genes in ICOS-L^High^ are shown in black, upregulated genes in ICOS-L^Low^ are shown in gray, and not differentially expressed genes in are shown in light blue. (D,E) Heatmap representations of the *z*-score expression values of the indicated DEGs (red, high expression; blue, low expression) from three independent donors (columns) for (D) cell surface markers and (E) secreted molecules. Gene expression values were normalized on the *B2M* and *RPL34* housekeeping genes.

To further explore the mechanisms by which these two phenotypically, morphologically and functionally distinct sub-populations of GM-CSF-DCs induced Tfh polarization, we performed RNA-sequencing (RNAseq) analysis of sorted GM-CSF-induced ICOS-L^High^ and ICOS-L^Low^ DCs (100,000 cells/sample, *n*=3 donors) (Fig. 6A). We detected 5118 significantly differentially expressed genes (DEGs) between the two sub-populations with an absolute fold change greater than two. More specifically, 2414 genes were upregulated in ICOS-L^High^ DCs and 2704 genes were upregulated in ICOS-L^Low^ DCs (Fig. 6B,C). Focusing on checkpoints and maturation markers, we observed that ICOS-L^Low^ DCs expressed *HLA-DRB1* and *HLA-DRA*, *CD276* (*B7H3*), and *CD40* at higher levels, as expected. ICOS-L^High^ DCs expressed transcripts associated with negative checkpoints, such as *CD274*, *PDCD1LG2* (which encodes PD-L2), *TNFRSF9* (which encodes 4-1BB), *IDO1* and *IDO2*, but also expressed transcripts encoding the positive checkpoint protein OX40 ligand (*TNFSF4*) and the maturation molecules CD70, CD80, CD83 and CD86 (Fig. 6D). Among secreted molecules, ICOS-L^High^ DCs preferentially expressed transcripts encoding IL-15, IL-7, IL-32 and the CCR7 ligand CCL19, whereas ICOS-L^Low^ DCs expressed higher levels of transcripts encoding IL-16, IL-1B, TNF-α, TRAIL (*TNFSF10*) and CCL4 (Fig. 6E). These data raised the question whether some of the DEGs were involved in the distinct Tfh polarization programs driven by the two GM-CSF-DC sub-populations.

### CD40-dependent Tfh1 polarization by GM-CSF-DCs

**Fig. 7.**
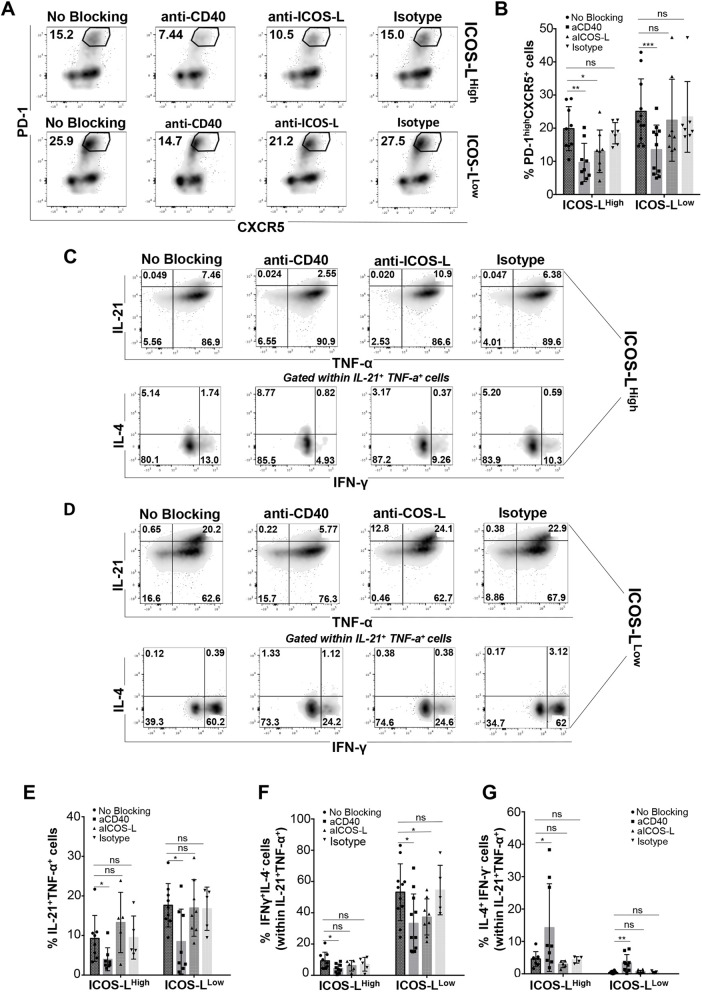
**CD40-dependent Tfh1 polarization by GM-CSF-DCs.** (A) PD-1^High^ CXCR5^+^ cells gated in total CD4^+^ T cells differentiated by either ICOS-L^High^ or ICOS-L^Low^ DCs incubated with blocking antibodies targeting CD40 or ICOS-L, or with the isotype control. One representative experiment is shown. Percentage of cells in the PD-1^high^ CXCR5^+^ population is shown on the plots. (B) Percentages of PD-1^High^ CXCR5^+^ cells from data as shown in A (aCD40, anti-CD40 blocking; aICOS-L, anti-ICOS-L blocking). Mean±s.e.m. from *n*=9. (C,D) Intracellular FACS staining for T cell cytokines induced either by (C) ICOS-L^High^ or (D) ICOS-L^Low^ DCs incubated with blocking antibodies against CD40 or ICOS-L, or with the isotype control, as indicated. One representative experiment is shown. IFN-γ and IL-4 production is gated within IL-21^+^ TNF-α^+^ cells. The percentage of cells in each gate is shown on the plots. (E–G) Percentages of (E) IL-21^+^ TNF-α^+^ cells, (F) IFN-γ^+^ IL-4^−^ cells gated in IL-21^+^ TNF-α^+^ cells and (G) IL-4^+^ IFN-γ^−^ cells gated in IL-21^+^ TNF-α^+^ cells from data as shown in C and D. Mean±s.e.m. from *n*=8. *P<0.05; **P<0.01; ***P<0.001; ns, not significant (two-tailed paired Student's *t*-test).

Both CD40 and ICOS-L have previously been shown to participate in the crosstalk between DCs and T cells (Ise et al., 2018; Leavenworth et al., 2015; Weber et al., 2015). Since their expression differed in the two sub-populations of GM-CSF-DCs, we performed CD40 and ICOS-L blocking experiments to evaluate their respective roles in Tfh differentiation. ICOS-L^High^ and ICOS-L^Low^ DCs were incubated for 60 min with blocking antibodies against human CD40 or ICOS-L before co-culture with allogeneic naive CD4^+^ T cells. CD40 blockade inhibited the induction of a PD-1^High^ CXCR5^+^ phenotype in T cells differentiated by both ICOS-L^High^ (no blocking, 19.8%±2.2; anti-CD40, 9.64%±1.9; mean±s.e.m.; *P*=0.0043, two-tailed, paired Student's *t*-test) and ICOS-L^Low^ DCs (no blocking, 25.1%±2.9; anti-CD40, 13.5%±2.2; *P*=0.0098, two-tailed, paired Student's *t*-test). ICOS-L blockade induced only a slight, but statistically significant, reduction in the formation of T_fh-like_ cells driven exclusively by ICOS-L^High^ DCs (13.0%±2.3 for ICOS-L^High^ and 22.4%±4.4 for ICOS-L^Low^) (Fig. 7A,B). We also compared the co-expression of cytokines produced by T cells that were previously polarized by each of the two populations of GM-CSF-DCs in the absence of either CD40 or ICOS-L signaling. CD40 blocking reduced the percentage of IL-21^+^ TNF-α^+^ cells in both conditions (ICOS-L^High^ no blocking, 9.3%±2.0; ICOS-L^High^ anti-CD40, 4.0%±1.0; ICOS-L^Low^ no blocking, 17.64%±1.82; ICOS-L^Low^ anti-CD40, 8.46%±2.90; mean±s.e.m.) as well as the percentage of IFN-γ^+^ IL4^−^ cells within the IL-21^+^ TNF-α^+^ T_fh-like_ cells (ICOS-L^High^ no blocking, 9.3%±1.8; ICOS-L^High^ anti-CD40, 4.3%±1.0; *P*=0.0315; ICOS-L^Low^ no blocking, 53.21%±5.46; ICOS-L^Low^ anti-CD40, 33.26%±5.65; *P*=0.0151, two-tailed, paired Student's *t*-test). Conversely, CD40 blocking increased the percentage of IL4^+^ IFN-γ^−^ cells in both conditions (ICOS-L^High^ no blocking, 4.6%±0.8; ICOS-L^High^ anti-CD40, 14.3%±4.5; *P*=0.0205; ICOS-L^Low^ no blocking, 0.41%±0.11; ICOS-L^Low^ anti-CD40, 3.36%±0.86; *P*=0.015, two-tailed, paired Student's *t*-test) (Fig. 7C–G). However, ICOS-L blocking did not affect the percentages of IL-21^+^ TNF-α^+^ and IL-4^+^ IFN-γ^−^ cells in any condition, except for a decrease in percentage of IFN-γ^+^ IL-4^−^ cells in the ICOS-L^Low^ DC condition (no blocking, 53.21%±5.46; anti-ICOS-L, 37.30%±4.08; *P*=0.0409, two-tailed, paired Student's *t*-test) (Fig. 7C–G). These data show a novel and prominent role for CD40 as a new molecule involved in human Tfh1 polarization.

### Positive correlation of GM-CSF-induced ICOS-L^Low^ DCs with Tfh1 cells in MTB infection and COVID-19

**Fig. 8.**
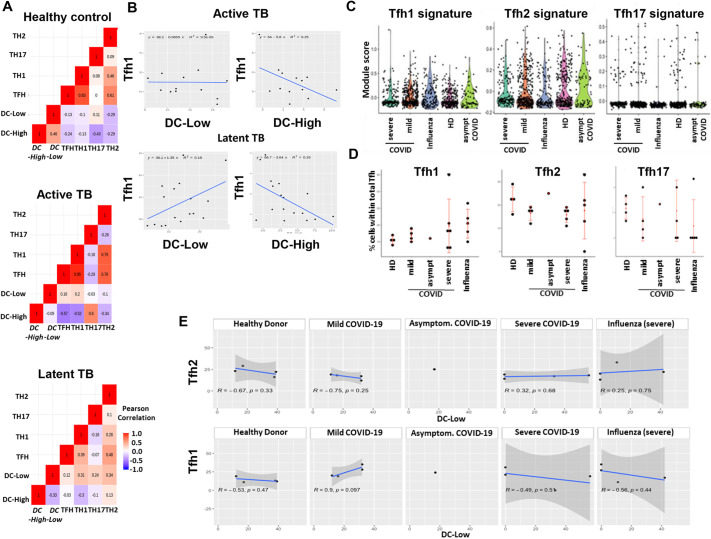
**GM-CSF-induced ICOS-L^Low^ DCs positively correlate with Tfh1 in MTB infection and COVID-19.** (A) Pearson pairwise correlation matrices of DC-High and DC-Low signatures, confronted with the indicated T effector cell signatures for each TB patient group. (B) Collinearity assessment of the Tfh1 signature with the DC-High and the DC-Low signatures within active TB and latent TB patients. Each dot represents a sample in the study data (Berry et al., 2010). A linear regression model was fitted to the data and is shown on the graphs. (C) Violin plots and single-cell data points for the expression level (module score) of Tfh1-, Tfh2- and Tfh17-related signatures expressed by all patient groups in the COVID-19 study data (Lee et al., 2020). Asympt, asymptomatic; HD, healthy donor. (D) Percentages of Tfh1, Tfh2 and Tfh17 cells within total Tfh cells in each patient group in the COVID-19 study data (Lee et al., 2020). Mean±s.e.m. No statistical significance was observed for any of these conditions. (E) Collinearity assessment of the corresponding signatures of Tfh2 and Tfh1 with the DC-Low subtype signature in all COVID-19 patient groups. Each dot represents a sample. A linear regression model was fitted to the data and is shown on the graph, with correlation coefficients and *P*-values indicated. Shaded regions denote the standard error.

Considering our results, we tested the hypothesis that GM-CSF-DC sub-populations are associated with the presence of Tfh1 cells in human infections. To explore a possible correlation between GM-CSF-DC signatures and Tfh subsets *in vivo*, we used two different clinical settings of infection: (1) whole-blood microarray data from MTB-infected patients (Berry et al., 2010) and (2) scRNAseq of peripheral blood mononuclear cells (PBMCs) from COVID-19 patients (mild, severe and asymptomatic) alongside severe influenza patients (Lee et al., 2020). Both studies included healthy controls. Based on our transcriptomic analysis of GM-CSF-DC sub-populations, we selected the top nine or ten genes expressed by each group to create in-house signatures for ICOS-L^High^ (DC-High) and ICOS-L^Low^ (DC-Low) GM-CSF-DCs (Table S3). First, we applied a deconvolution method to assess the presence of all major immune cell types in the microarray data of whole blood from patients with either active or latent tuberculosis (TB) and from healthy controls (Fig. S6A), which allowed us to make comparisons between samples and cell types (Fig. S6B). Using Pearson pairwise correlation matrices of DC-High and DC-Low signatures confronted with T effector (Th1, Th2, Th17 and Tfh) cell signatures (Table S2) we observed that Tfh cells were significantly associated only with DC-Low in active and latent TB patient samples, but not in healthy controls (Fig. 8A). Next, we focused on a possible association with the diverse Tfh subsets. Interestingly, we identified a positive correlation between DC-Low and Tfh1 signatures in latent TB (r=0.44), but not in active TB, and a negative correlation between DC-High and Tfh1 in both latent TB (r=0.54) and active TB (r=0.50) (Fig. 8B). Neither DC-High nor DC-Low signatures had any significant positive correlation with other subsets of Tfh cells (Tfh2 or Tfh17; Fig. S6C,D) emphasizing the important relationship between the sub-population of ICOS-L^Low^ GM-CSF-DCs and Tfh1 cells, which might be needed to provide a protective environment in latent TB.

In addition, we tried to validate our new experimental findings in a clinical setting of COVID-19 (Fig. S7A). We sought to determine whether we could detect any positive correlation between Tfh1 cells and the DC-Low signature. First, the use of CD3 and CD4 as universal markers for the identification of T cells allowed us to detect them in all the disease groups. We also recovered the Th cell subsets using in-house constructed signatures (Fig. S7B; Table S2). Th1, Th2 and Tfh signatures were observed in sufficient numbers in all patient groups, whereas very few cells expressed the Th17 signature. Interestingly, mild COVID-19 patients revealed higher Th1 numbers as compared to both severe COVID-19 patients and influenza patients. The same trend was observed for both Th2 and Tfh cells, suggesting a more efficient adaptive immune response in mild COVID-19 patients (Fig. S7C). Since the focus of our study was on Tfh subsets, we used our in-house constructed signatures to identify Tfh1, Tfh2 and Tfh17 cells within the Tfh cluster. The distribution of the corresponding module scores revealed higher positivity for Tfh1 cells as compared to both Tfh2 and Tfh17 cells (Fig. 8C). This observation was additionally confirmed by statistical comparison of the percentage of positive cells for each signature among total Tfh cells at the patient level. Tfh1 cells were found to be increased mostly in mild COVID-19 patients (Fig. 8D); however, the small cohort size did not allow the conduction of statistically significant tests. In parallel, we looked for the two DC signatures in cells positive for CD11c (also known as ITGAX) and BDCA1 (also known as CD1C) (Fig. S7D). We could not detect any cells expressing the DC-High signature in all disease groups, whereas cells displaying the DC-Low signature were present (Fig. S7D). The violin plot representation revealed that DC-Low-positive cells could mostly be detected in mild COVID-19 patients, even if the ratio within the total DC cell population was almost the same in all disease groups (Fig. S7E). The estimation of both the DC and Tfh subsets is defined by the ratio detected in each patient. Finally, we sought to correlate the percentages of Tfh subsets and DC-Low cells in each disease severity group by applying a Pearson correlation test. The only statistically significant (*P*<0.1) positive correlation we detected was between the Tfh1 and DC-Low signatures in mild COVID-19 patients (Fig. 8E). This observation might be explained by the better response of these patients, which requires a more activated Tfh1 profile for a more efficient differentiation of plasma cells. The presence of ICOS-L^Low^ GM-CSF-DCs might be necessary for inducing efficient Tfh1 responses.

## DISCUSSION

In this study, we provide evidence for the key role of GM-CSF-DCs and CD40 in inducing polarization of human naive CD4^+^ T cells into bona fide Tfh1 cells. BCL6 has previously been shown to be the lineage-defining transcription factor of Tfh cells, regulating their functional properties (Hatzi et al., 2015; Liu et al., 2012; Nance et al., 2015; Choi et al., 2015). Co-expression of BCL6 with other Th transcription factors might imprint Tfh cells with additional functions playing a crucial role in regulating B-cell induced immunity. More specifically, it has been shown that viral infections promote TBET expression in Tfh cells, which contributes to IFN-γ secretion and the type of antibodies produced by plasma cells (Weinstein et al., 2018; Sheikh et al., 2019). Even transient TBET expression is sufficient to make Tfh cells secrete IFN-γ for long time (Fang et al., 2018). We have previously shown that TSLP-activated DCs induce polarization of human naive CD4^+^ T cells into Tfh cells expressing high levels of GATA3 and secreting significant amounts of IL-4 (Pattarini et al., 2017). More recently, a new study has provided evidence for the existence of a population of Tfh cells, referred to as TFH13, that has an unusual cytokine profile (IL-13^high^ IL-4^high^ IL-5^high^ IL-21^low^) and co-expresses BCL6 and GATA3, affecting high-affinity IgE production and subsequent allergen-induced anaphylaxis (Gowthaman et al., 2019). In support of these findings, we demonstrated that GM-CSF-DC-induced Tfh cells secreted large amounts of IFN-γ and expressed high levels of TBET, confirming a strong relationship between transcription factor expression and subsequent cytokine secretion. However, the identification of two distinct sub-populations of GM-CSF-DCs with different functions in T cell polarization raised the question of whether cytokine production can be dissociated from transcription factor expression. ICOS-L^High^-induced Tfh cells secreted higher levels of IL-4 and much lower amounts of IFN-γ as compared to ICOS-L^Low^-induced Tfh cells, but without any major differences in the expression of either GATA3 or TBET. These observations suggest that the expression of transcription factors might not be always sufficient to identify distinct Tfh subsets, since the cytokine profile could reflect a different polarization program. This is not very surprising, because interactions between Tfh cells and GC B cells or Tfh cells and DCs can lead to cytokine secretion that can signal through the Tfh cells, leading to the expression of transcription factors aside from GATA3 and/or TBET. We cannot exclude the possibility that our *in vitro* system might lead to transient activation of other transcription factors. Further *in vitro* and *ex vivo* studies are needed to better understand the role of transcription factors in the functions of distinct Tfh subsets.

GM-CSF was among the first cytokines shown to efficiently promote DC development *in vitro* from monocytes and hematopoietic progenitor cells (Inaba et al., 1992; Sallusto and Lanzavecchia, 2018; Caux et al., 1996, 1992). It is considered to be a regulator of granulocyte, monocyte and DC lineages at all stages of maturation, with effects on cytokine secretion, cytotoxicity and antigen-presentation capability (Hamilton, 2008; Hornell et al., 2003; van de Laar et al., 2012). In our study, we used single-cell approaches to show that GM-CSF induces the diversification of human blood DCs into distinct sub-populations with different phenotypes, morphologies, transcriptomic signatures and functions. Interestingly, the function of total GM-CSF-DCs in activating CD4^+^ T cells was similar to the effects of the ICOS-L^Low^ DC sub-population, showing that the latter plays a dominant role in T cell polarization. Previous studies of bulk GM-CSF-DCs may have been biased by the function of dominant DC sub-populations, masking an underlying functional heterogeneity.

CD40 expression on DCs has been to be necessary for promoting survival, cytokine production and activation of naive T cells (Grewal and Flavell, 1996; Stout and Suttles, 1996). CD40 is known to induce secretion of IL-12, resulting in enhanced Th1 immune responses (Kelsall et al., 1996; Macatonia et al., 1995; Dubois et al., 1998), but there is no direct link with other Th differentiation programs. Interestingly, the reduced incidence of Tfh cells in patients with immune deficiencies caused by mutations in CD40 ligand (Bossaller et al., 2006) has raised questions about a potential role of CD40 in both the generation and maintenance of Tfh cells. In our study, we showed by both transcriptomic and single-cell protein approaches that CD40 was highly expressed by ICOS-L^Low^ DCs, the sub-population of GM-CSF-DCs that induced strong Tfh1 responses. Blocking of CD40 in GM-CSF-DCs resulted in reduced frequencies of the common Tfh phenotypic markers PD-1 and CXCR5, together with decreased levels of their key effector cytokine, IL-21. Additionally, inhibition of CD40 expression induced a dramatic decrease in IFN-γ by IL-21-producing Tfh cells, favoring the secretion of IL-4. Taken together, these data suggest that the expression of CD40 by GM-CSF-DCs is involved in both Tfh and Tfh1 polarization programs. Its absence might allow other co-stimulatory molecules to dominate, creating a microenvironment with different effects on the type of Tfh differentiation. CD40 could be considered as a new therapeutic target for the manipulation of Tfh and Tfh1 cells.

The identification of MTB infection-specific IL-21^+^ IFN-γ^+^ Tfh1-like cells (Li et al., 2016), together with a decrease in frequency of Tfh cells detected in the blood of active TB patients (Kumar et al., 2014; Slight et al., 2013), raises the question whether Tfh1 cells display immune regulatory function. Additionally, the protective role of GM-CSF has already been shown in several MTB infection studies (Gonzalez-Juarrero et al., 2005; Wang et al., 2002; Bryson et al., 2019). We observed a strong positive correlation between DCs displaying the ICOS-L^Low^ GM-CSF-DC signature and Tfh1 cells, exclusively in patients with the latent form of MTB infection. These results validate our *in vitro* findings about the dependence of Tfh1 differentiation on a specific subset of GM-CSF-DCs and open new perspectives for the mechanism used by GM-CSF to induce protective effects in MTB infection. Recent studies have demonstrated a strong positive correlation between circulating Tfh1 cells and the magnitude of viral specific antibodies in COVID-19 convalescent patients, highlighting a new crucial role for Tfh1 cells in the fight against viral infections (Juno et al., 2020; Gong et al., 2020; Zhang et al., 2020a,b). The severity of COVID-19 infection might be also associated with the function of Tfh1 cells, since it has recently been shown that in active severe COVID-19 patients there is a loss of BCL6^+^ Tfh cells and GCs, together with an increase in TBET^+^ Th1 cells (Kaneko et al., 2020). The huge production of cytokines in severe COVID-19 patients might block GC development, inhibiting the transformation of Th1 cells into Tfh cells (Kaneko et al., 2020). Taking advantage of publicly available data (Lee et al., 2020), we identified a positive correlation between Tfh1 cells and ICOS-L^Low^ GM-CSF-DCs exclusively in mild COVID-19 patients. Those patients are characterized by a better response to SARS-COV-2; by avoiding a strong cytokine storm, their immune system might allow an efficient generation of Tfh cells and GC development. Additionally, their Tfh responses could be enhanced by the presence of DCs displaying a specific activation profile favoring the polarization of Tfh1 cells. SARS-CoV-2 can make host cells secrete GM-CSF, which could enhance the ability of DCs to better prime naive T cells during antigen-specific immune responses (Becher et al., 2016; Guilliams et al., 2013). Additionally, the capacity of GM-CSF to maintain pulmonary function and lung cell-mediated immunity, together with its protective functions in mouse models of influenza, suggest that GM-CSF administration is a possible therapy against COVID-19 (Lang et al., 2020; Trapnell et al., 2019; Rösler and Herold, 2016). Indeed, several clinical trials are already ongoing (Lang et al., 2020).

## MATERIALS AND METHODS

### Human subjects

Apheresis blood from healthy human blood donors was obtained from Etablissement Francais du Sang (French Blood Establishment) after written informed consent and in conformity with Institute Curie and Research Institute Saint-Louis ethical guidelines. Gender identity and age of anonymous donors were not available, but all donors were between 18 and 70 years old (age limits for blood donation in France).

### Purification of DCs and naive CD4^+^ T lymphocytes from blood

Peripheral blood mononuclear cells (PBMCs) were isolated by centrifugation on a density gradient (Lymphoprep, 07801, StemCell Technologies) following the manufacturer's protocol. Primary blood DCs were purified according to an established protocol (Alculumbre and Pattarani, 2016). In brief, total PBMCs were enriched in DCs using the EasySep Human Pan-DC Pre-Enrichment kit (StemCell Technologies). Enriched DCs were then sorted to obtain 98% purity on an MoFlo Astrios cell sorter (Beckman Coulter) or FACS ARIA III (BD Technologies), as lineage^−^ (CD3, CD14, CD16, CD56, CD20 and CD19; FITC anti-human CD3e, BD, 555339; FITC anti-human CD14, Miltenyi Biotec, 130-080-701; FITC anti-human CD16, BD, 347523; FITC anti-human CD56, BioLegend, 318304; FITC anti-human CD20, BD, 555622; FITC anti-human CD19, Miltenyi Biotec, 130-113-168), CD4^+^ (BV-650 anti-human CD4, BioLegend, 317436), CD11c^+^ (PeCy7 anti-human CD11c, Biolegend, 337216) and CD1c^+^ (PerCP-eFluor710 anti-human CD1c, also known as BDCA1, Thermo Fisher Scientific, 46-0015-42).

After enrichment from total PBMCs using the CD4^+^ T cell isolation kit (StemCell Technologies), naive CD4^+^ T cells were magnetically isolated. Purity was at least 95%.

### Flow cytometry analysis

Antibodies were titrated on the relevant human PBMC population, and matched isotypes controls were used at the same final concentrations. For ICS, CD4^+^ T cells were stimulated with 100 ng/ml PMA, 500 ng/ml ionomycin and 1:1000 brefeldin A (eBioscience, 00-4506-51) for 4 h. When cells were sorted before intracellular staining, they were cultured overnight in X-VIVO medium (Lonza) at 10^6^ cells/ml before PMA and ionomycin stimulation. To exclude dead cells, CD4^+^ T cells were stained using the LIVE/DEAD Fixable Yellow dead cell stain kit, following the manufacturer's instructions (Thermo Fisher Scientific, L34959). Cells were fixed and permeabilized using IC Fix and Permeabilization buffers (both eBioscience). Intracellular cytokines were revealed with fluorescently conjugated antibodies against IL-21 (PE anti-human IL-21, BioLegend, 513004; 1:20), TNF-α (Alexa Fluor 700 anti-human TNF-α, BioLegend, 502928; 1:20), IL-4 (APC anti-human IL-4, Thermo Fisher Scientific, 17-7049-42; 1:10), IFN-γ (PeCy7 anti-human IFN-γ, Thermo Fisher Scientific, 25-7319-82; 1:40) and IL-17A (BV-650 anti-human IL-17A, BD, 563746; 1:10) or matched isotype controls (eBioscience) and acquired on a LSR Fortessa instrument (BD Biosciences). For transcription factor intranuclear staining, dead cells were first stained with a yellow dye (LIVE/DEAD Fixable Yellow dead cell stain, Thermo Fisher Scientific), followed by staining using fluorescently conjugated antibodies against PD-1 (BV-711 anti-human PD-1, Biolegend, 329928) and CXCR5 (BV-421 anti-human CXCR5, BD, 562747). After fixation and permeabilization using the FOXP3 IC buffer kit (eBioscience), cells were stained with antibodies to detect BCL6 (APC anti-human BCL6, BD, 561525), TBET (PerCP-Cy5.5 anti-human T-BET, Thermo Fisher Scientific, 45-5825-82), GATA3 (PeCy7 anti-human GATA3, Thermo Fisher Scientific, 25-9966-42), RORC (PE anti-human RORγT, Thermo Fisher Scientific, 12-6988-82), C-MAF (PE anti C-MAF, Thermo Fisher Scientific, 12-9855-42) or SAP (eFluor 660 anti-SAP, Thermo Fisher Scientific, 50-9787-42), and acquired on a LSR Fortessa instrument. As a control for intracellular staining of transcription factors, cells were stained with matched isotype controls at the same concentration as the transcription factor antibodies. The fluorescence obtained in each channel and in each population in the presence of the isotype control antibody (fluorescence minus one, FMO) was subtracted from the fluorescence obtained by the specific staining of transcription factors in each population. For the phenotypic analysis of GM-CSF-DCs and Medium-DCs in Fig. 4A,B, the following antibodies were used: APC eFluor 780 anti-human HLA-DR (Thermo Fisher Scientific, 47-9956-42), FITC anti-human CD40 (BD, 555588), PE anti-human ICOS-L (Thermo Fisher Scientific, 12-5889-42), PeCy5 anti-human CD86 (Thermo Fisher Scientific, 15-0869-42), PeCy7 anti-human CD80 (BioLegend, 305218), PerCP-eFluor 710 anti-human CD274 (PD-L1; Thermo Fisher Scientific, 46-5983-42), APC/Cy7 anti-human CD25 (BD, 557753) and PE anti-human nectin-II (BioLegend, 337410). For all flow cytometry-related analysis, we used the BD Fortessa Machine but in two different sites with differences in their settings, explaining possible variations in the way similar staining appears. FlowJo software (TreeStar) was used for flow cytometry analysis.

### DC activation

DCs were cultured in RPMI 1640 medium with GlutaMAX (Life Technologies) containing 10% fetal calf serum (Hyclone), 100 U/ml penicillin-streptomycin (Gibco), MEM non-essential amino acids (Gibco) and 1 mM Na pyruvate (GIBCO). DCs were cultured at 10^6^ cells/ml in flat-bottom plates for 24 h or 48 h in the presence of 50 ng/ml rhGM-CSF (Prospec) (unless specified otherwise) or 100 ng/ml ultrapure LPS (InvivoGen).

### DC and T cell co-culture

For co-culture, activated DCs were washed twice in PBS and put in culture with allogeneic naive CD4^+^ T cells (10^4^ DCs and 5×10^4^ T cells) in X-VIVO 15 medium (LONZA) for the indicated time (4 or 6 days). For co-culture, CD4^+^ T cells were freshly purified from PBMCs the day after DC purification or 2 days later, depending on the experimental condition. A single DC donor was paired exclusively with a single CD4^+^ T cell donor to perform each co-culture experiment. DCs were stimulated either with rhGM-CSF or LPS for 24 h to activate total cells, or only with rhGM-CSF for 48 h or 72 h to induce the emergence of two different sub-populations. After 2 days of activation, sub-populations of DCs were electronically sorted (ARIA III, BD) based on the expression of ICOS-L and CD40. Total activated DC or both sub-types of GM-CSF-DCs were put in co-culture with allogeneic naive CD4^+^ T cells with the same ratio as mentioned above.

### Blocking experiments

DCs were incubated at 37°C with 50 ng/ml anti-human CD40 antibody (Ultra-LEAF purified anti-human CD40, BioLegend, 668104), 50 ng/ml anti-human ICOS-L [Ultra-LEAF purified anti-human CD275 (B7-H2, B7-RP1, ICOS-L), BioLegend, 329808] or 50 ng/ml of the corresponding isotype control (BioLegend). After 60 min, CD4^+^ naive T cells were added to the culture. Blocking antibodies were present for the duration of the co-culture. At the indicated time points, cells were either subjected to fluorescence-activated cell sorting (FACS), used for surface or intracellular staining, or washed and reseeded at 10^6^ cells/ml and re-stimulated with aCD3/aCD28 beads (LifeTech) for 24 h, after which supernatants and cells were collected for analysis. At day 4, T cells were counted and analyzed for the induction of a Tfh profile. At day 6, T cells were counted and analyzed for their cytokine production by FACS.

### Co-culture of T cells and B cells

After 4 days of co-culture with total GM-CSF-DCs, CD4^+^ T cells were FACS sorted as PD-1^High^ CXCR5^+^ (T_fh-like_ cells) or PD-1^Low^ CXCR5^+^ (T_Low_ cells). Allogeneic PBMCs were thawed and, after a round of human memory B cell enrichment, memory B cells were magnetically sorted using the EasySep Human Memory B cell isolation kit (StemCell Technologies). T cells and B cells were co-cultured in X-VIVO medium in round-bottom plates (2.5×10^5^ T cells and 2.5×10^5^ memory B cells). At day 10 of culture, cells were harvested for FACS analysis.

### Cytokine quantification

Cytokines were quantified in supernatants using ELISAs for IL-21 (BioLegend) and CXCL13 (R&D Systems) or cytometric bead array (CBA) flex sets for IL-2, IL-3, IL-4, IL-5, IL-9, IL-10, IL-13, IL-17A, GM-CSF, TNF and IFN-γ (BD Biosciences), following the manufacturer's protocol. CBA analysis used FCAP Array v3 software (BD Biosciences).

### Transcriptomic analysis

Samples were sequenced at QuickBiology, Pasadena, CA. Briefly, RNA integrity was checked using an Agilent Bioanalyzer 2100. Libraries for RNA-Seq were prepared according to the KAPA stranded mRNA-Seq poly(A)-selected kit with 201–300 bp insert size (KAPA Biosystems, Wilmington, MA) using 250 ng total RNA as input. Library quality and quantity were analyzed using an Agilent Bioanalyzer 2100 and Life Technologies Qubit 3.0 Fluorometer. Then, 150 bp paired-end reads were sequenced on an Illumina HiSeq 4000 (Illumnia Inc., San Diego, CA). The reads were first mapped to the hg19 UCSC transcript set using Bowtie2 version 2.1.0, and the gene expression level was estimated using RSEM v1.2.15. Downstream analyses were performed using R (v3.6.0) and DESeq2 package (v1.26.0). DEGs were determined with an absolute log fold change threshold at 2 and an adjusted *P*-value below 0.01.

### Microarray data analysis

We loaded the normalized dataset using GEOquery R package, and GSE19444 as studyID (Berry et al., 2010). We selected the top nine DEGs between ICOS-L^High^ DCs and ICOS-L^Low^ DCs from our bulk transcriptomic analysis and used the T subtypes signatures previously used. We estimated the fraction of detected cell types in the samples using Quanti Seq deconvolution algorithm from the immunedeconv R package. For each DC and CD4^+^ T cell subtype, we calculated a signature score as the median expression values of the set of genes of a given signature. To assess the relationships between DC subsets and Tfh–Th1 polarization, we plotted the corresponding subtype signature scores and fitter the scatter graphs using linear regression [lm()] function in R.

### scRNAseq data analysis of COVID-19 and severe influenza patients

We performed the single-cell data analysis in R (version 4.1) using the Seurat R toolkit package. Associated metadata of the whole analyzed cells were loaded and added to the Metadata slot of the Seurat object. First, we created a subset Seurat object containing only CD4^+^ T cells. Next, we defined Th cell subsets (Th1, Th2, Th17 and Tfh) using as input our in-house signature genes, and constructed scores for each Th subset for each individual cell using the ‘AddModuleScore’ Seurat function, setting both the number of bins and control genes to *n*=100. A similar procedure was applied to retrieve CD1c^+^ DCs, using the expression levels of canonical markers (positive values for *CD1C* and *ANPEP* genes, and absence of expression of *THBD* gene). Similarly, we applied both DC-Low (ICOS-L^Low^ GM-CSF-DC) and DC-High (ICOS-L^High^ GM-CSF-DC) signatures to the DCs and followed the same procedure to estimate the percentage of DCs for each patient. The analysis code for this work is available on Github (https://github.com/MelissaSaichi/Tfh_GMCSF-DC).

### scRNAseq, quality control and pre-processing of expression matrices

We performed scRNAseq analysis of naive CD4^+^ T cells stimulated with Medium-DCs, LPS-DCs or GM-CSF-DCs using Chromium 10X technology (10X Genomics). Cell Ranger software (10X Genomics) was used to generate FASTQ files and align them to the GRCh38 human reference genome. The expression matrix datasets were loaded on R (version 4.0.0), and the whole analysis was performed using the Seurat package (version 3.2.0 https://github.com/satijalab/seurat). The three datasets corresponding to each condition were analyzed separately. For each sample, only cells expressing at least 100 genes were retained, in order to exclude debris cells. Pre-processing steps were applied to remove cells with fewer than 100 expressed genes or having more than 20% of mitochondrial transcripts. Upper cutoffs of 8000 and 90,000 were manually set for the nFeatures and nUMI, respectively, for each sample. In contrast, lower cutoffs of 3500 and 20,000 were set for nFeatures and nUMI, respectively. Normalization to 10,000 reads, centering and scaling were sequentially applied on the expression matrices to correct for sequencing depth variability.

### Downstream analysis of scRNAseq data

We used the MAGIC (Markov affinity-based graph imputation of cells; https://github.com/KrishnaswamyLab/MAGIC) imputation method to denoise data and correct for the dropouts, and stored the corrected expression matrices into the ‘imputed’ slot of Seurat objects. To construct Th-related gene signatures, we used the ‘AddModuleScore’ Seurat function using ten control genes and four bins. Cells that overexpressed or underexpressed a given module were attributed positive or negative scores, respectively. This strategy allowed us to identify cells co-expressing the genes used to identify the different cell types.

The analysis code is deposited at Github: https://github.com/MelissaSaichi/Tfh_GMCSF-DC.

### Dimension reduction and clustering of scRNAseq data

The normalized count matrices were used to identify highly variable genes within each dataset separately using the ‘mvp’ method implemented in the ‘FindVariableFeatures’ function of Seurat. For each sample, PCA dimension reduction was applied on the top 3000 genes, and the first 20 principal components (PCs) were used for further steps, including cell community detection, clustering and non-linear dimension reduction. Cell clusters were identified using a shared nearest neighbor (SNN) clustering algorithm, which consists of a calculation of the *k* nearest neighbors (*k*=30) followed by identification of cell communities (clusters) with a resolution parameter of 0.4. A non-linear dimensionality reduction method – uniform manifold approximation and projection (UMAP) – was used to explore and visualize the datasets, given as input the top 20 principal component genes.

### Imaging flow cytometry

Samples were acquired using ImageStreamX MkII technology (Amnis/Luminex). Laser power was set as 25 mW for the 405 nm laser, 80 mW for the 488 nm laser and 200 mW for the 561 nm laser to excite DAPI viability dye in channel 7 (430–505 nm filter), anti-CD40–FITC in channel 2 (480–560 nm filter) and anti-ICOS-L–PE in channel 3 (560–595 nm filter), respectively. Channels 1 (430–480 nm filter) and 9 (570–595 nm filter) were both used to collect brightfield images. Data were acquired with 60× magnification, a 7 µm core size and low flow rate. Analysis was performed with IDEAS software (Luminex) to calculate brightfield circularity (arbitrary unit) for each live population of interest selected based on the mean fluorescence intensities (MFIs) of CD40 and ICOS-L.

### Statistical analysis

Statistical analysis was performed using the Prism software v7 (GraphPad). Two-tailed, paired Wilcoxon tests or two-tailed, paired Student's *t*-tests were applied to compare two groups. Mann–Whitney test was used for non-paired analysis. Significance was retained for *P*<0.05. *P<0.05, **P<0.01, ***P<0.001, ****P<0.0001.

## Supplementary Material

10.1242/joces.260298_sup1 Supplementary information
