# Supplementary material for: GM-CSF-activated human dendritic cells promote type 1 T follicular helper cell polarization in a CD40-dependent manner
Source: J Cell Sci. 2022 Nov 11;135(21):jcs260298. doi: 10.1242/jcs.260298 (PMC9687542; doi:10.1242/jcs.260298)
Supplement: Supplementary information [file joces-135-260298-s1.pdf]

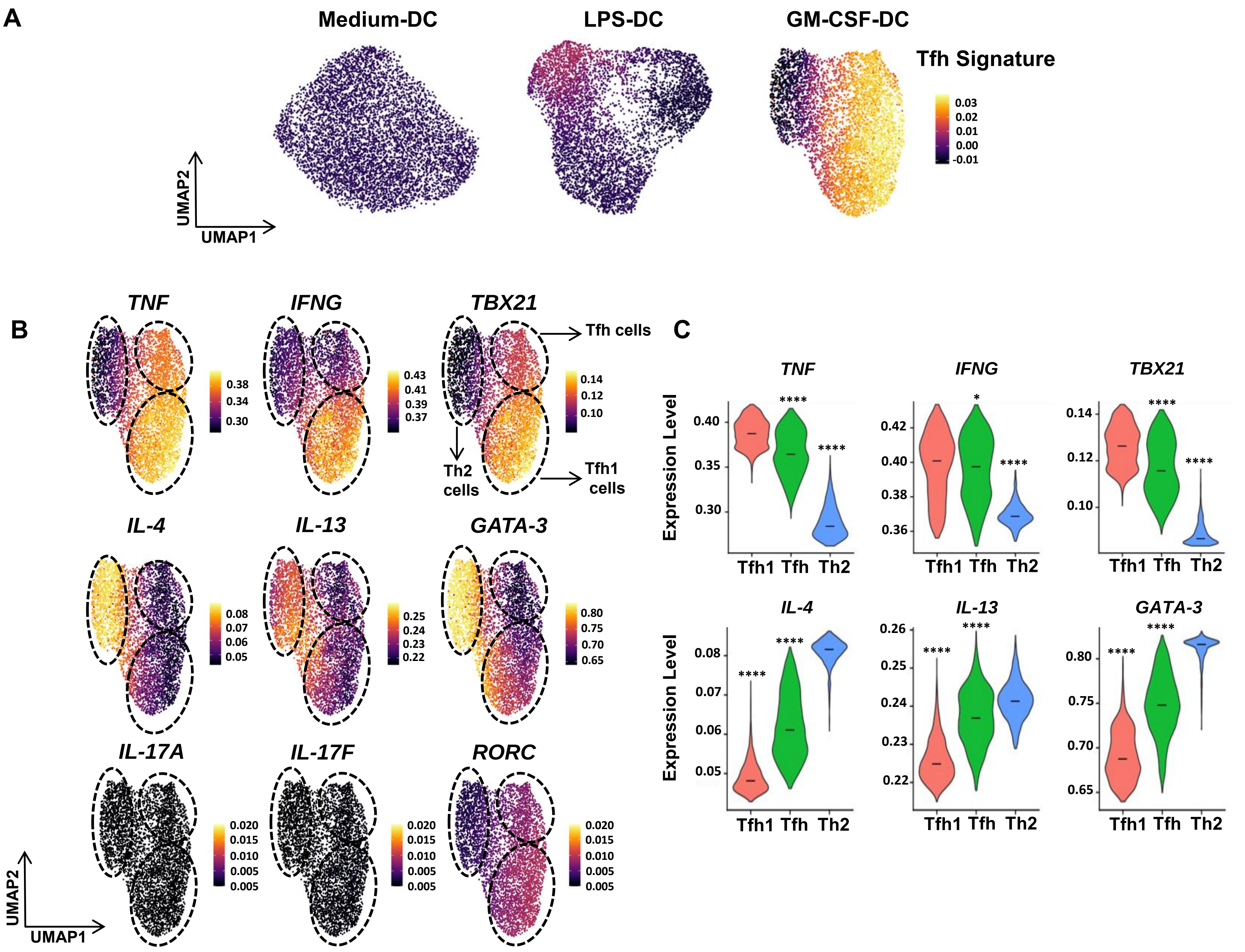

Fig. S1. Expression of Th-related genes by CD4$^+$ T cells activated by GM-CSF-DC, LPS-DC and Medium-DCType. (A) UMAP representation of the Tfh signature score of differentiated CD4$^+$ T cells in the three following conditions of DC activation: Medium-DC, GM-CSF-DC and LPS-DC. Signature scores were calculated by the co-expression of the Tfh related genes (BCL6, PD-1, CXCR5, IL-21). **(B)** Corrected expression values of the Th1, Th2 and Th17 related genes respectively, represented on the UMAP plot of the CD4$^+$ T cells differentiated by GM-CSF-DC. Th1 and Tfh related genes are co-expressed only by the CD4$^+$ T cells differentiated by GM-CSF-DC condition. **(C)** Violin plot representation for the expression level of Th1 and Th2 related genes in Tfh (including pure-Tfh and Tfh1)- and Th2-enriched clusters. Asterisks above a violin plot show statistical differences as compared to the violin plot condition without any asterisks. *P < 0.05, **P < 0.01, ***P < 0.001

Journal of Cell Science • Supplementary information

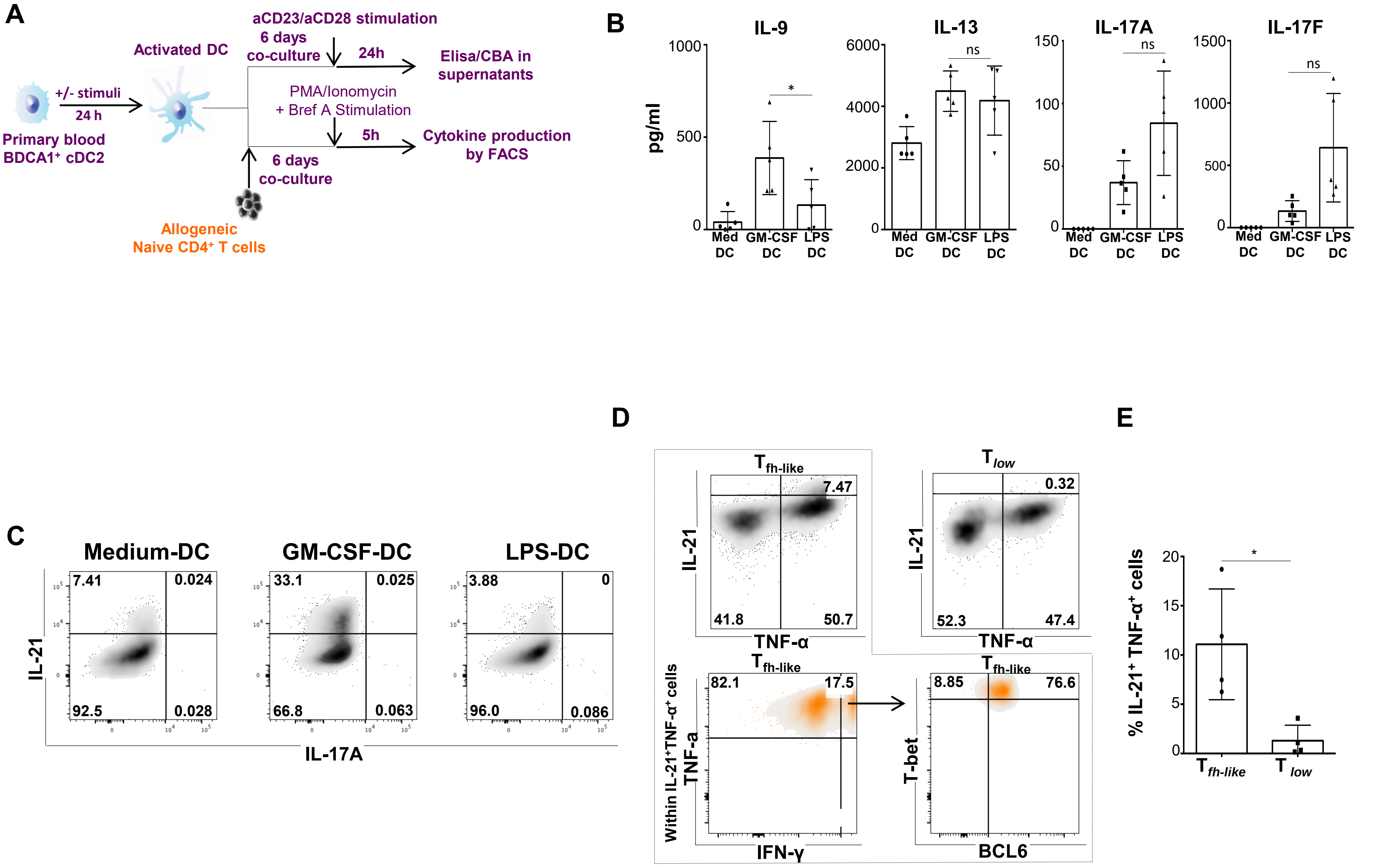

Fig. S2. Cytokine profile of CD4^{+} T cells differentiated by GM-CSF-DC. (A) Experimental approach for the measurement of cytokines produced by CD4^{+} T cells differentiated by activated DC. DC were activated for 24h in the presence of GM-CSF, LPS or only Medium, followed by 6 days co-culture with allogeneic naive CD4^{+} T cells. At day 6, T cells were stimulated either with aCD3/ aCD28 beads for additional 24h to measure cytokine secretion in the supernatants or with PMA/ionomycin and brefeldin-A for 4h, permeabilized and stained for the detection of several cytokines at a single cell level by flow cytometry. **(B)** CBA (IL-9, IL-13, IL-17A, IL-17F) assay for the measurement of cytokines in the supernatants of T cells in each condition of DC activation. Data are mean ± SEM from 5 independent experiments (n=5). **(C)** Intracellular staining for the expression of IL-21 and IL-17A by flow cytometry in T cells, one representative experiment (n=6). **(D)** Intracellular staining for the expression of IL-21, TFN- , IFN-γ, BCL6 and T-BET by sorted Tfh-like and Tlow cells in GM-CSF-DC condition for one representative experiment (n=4). **(E)** Percentages of IL-21^{+}TNF-α^{+} cells from data as shown in D for four independent experiments (n=4), *, P < 0.05; **, P < 0.01; ***, P < 0.001, by Wilcoxon or Student's t test.

Journal of Cell Science • Supplementary information

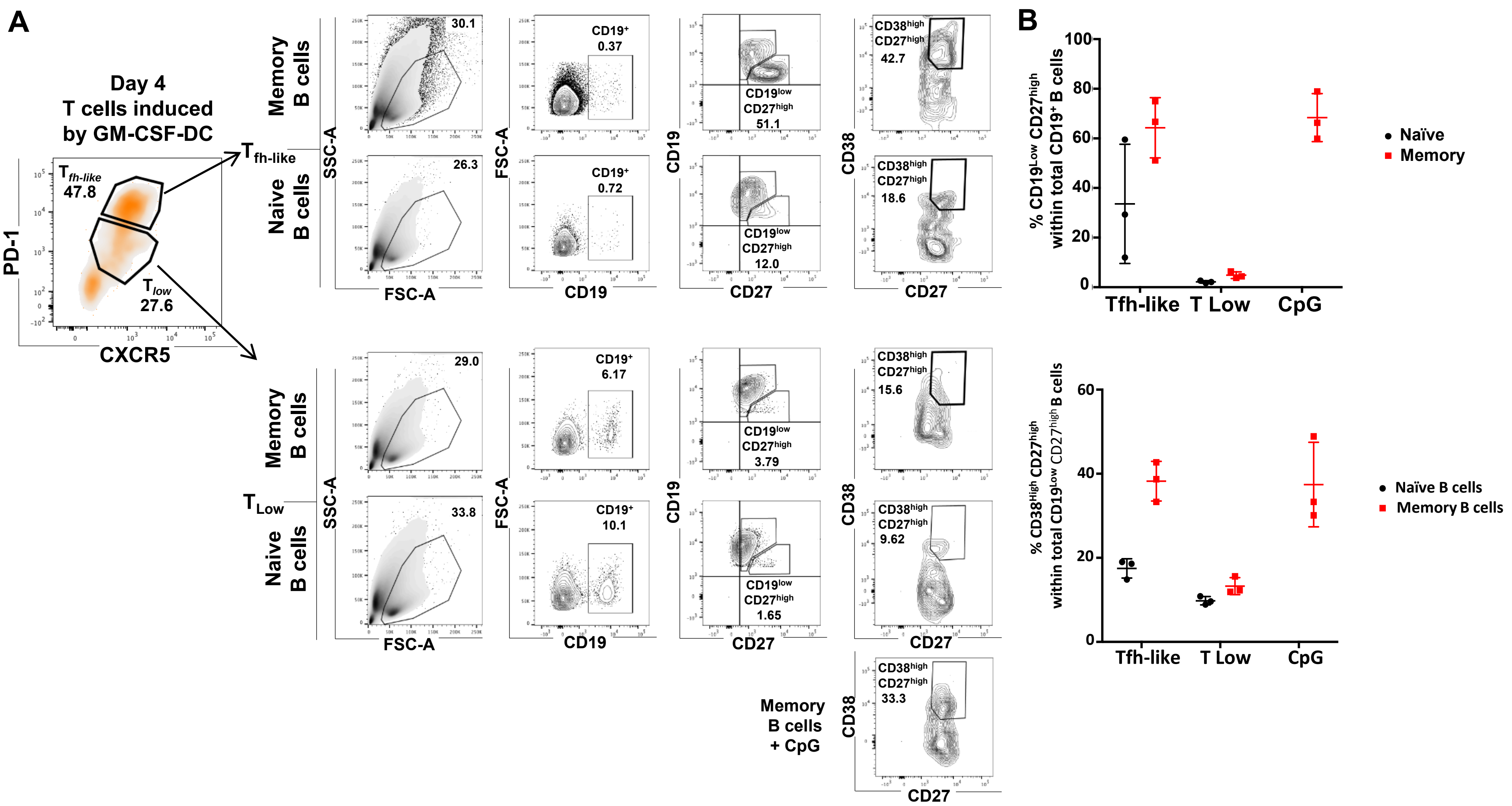

Fig. S3. Plasma Cell differentiation induced by GM-CSF-DC-activated Tfh cells. (A) Tfh-like and Tlow cells were sorted after four days of co-culture of naive $CD4^+$ T cells and GM-CSF-DC based on the expression of PD- 1 and CXCR5. T cells were then put in co-culture with allogeneic memory or naive B cells for 10 days. Extracellular staining for the expression of CD19, CD27 and CD38 for one representative experiment (n=3). **(B)** Percentages of $CD19^{low}CD27^{high}$ cells gated in total $CD19^+$ cells and $CD38^{high}CD27^{high}$ gated in $CD19^{low}CD27^{high}$ cells from data as shown in A (n=3), *, P < 0.05; **, P < 0.01; ***, P < 0.001, by Wilcoxon or Student's t test.

Journal of Cell Science • Supplementary information

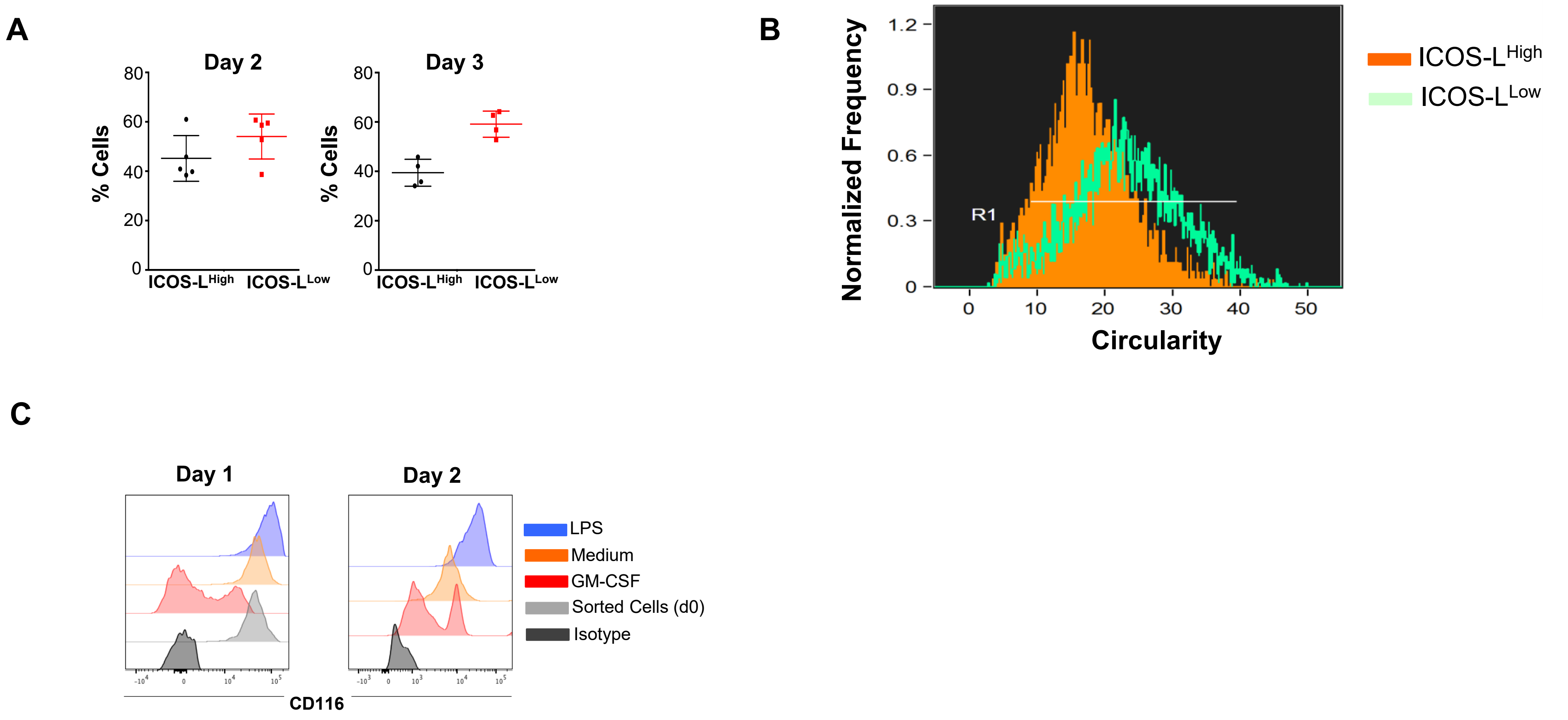

Fig. S4. Morphological and phenotypic characterization of GM-CSF-DC. (A) Percentages of recovered ICOS-L^{High} and ICOS-L^{Low} DC after 2 and 3 days of activation with GM-CSF from data as shown in FIG 4D (n=5). **(B)** Histograms comparing the circularity levels of both ICOS-L^{High} and ICOS-L^{Low} GM-CSF-DC at day 2 by Imaging Flow Cytometry approach (n=3). **(C)** Expression of CD116 by FACS of human DC right after sorting (light grey histogram) or DC activated with LPS (blue histogram) or GM-CSF (red histogram) for 48h as compared to non-activated DC (orange histogram) and isotype control (dark grey histogram) for one representative experiment (n=4), *, P < 0.05; **, P < 0.01; ***, P < 0.001, by Wilcoxon or Student’s test.

Journal of Cell Science • Supplementary information

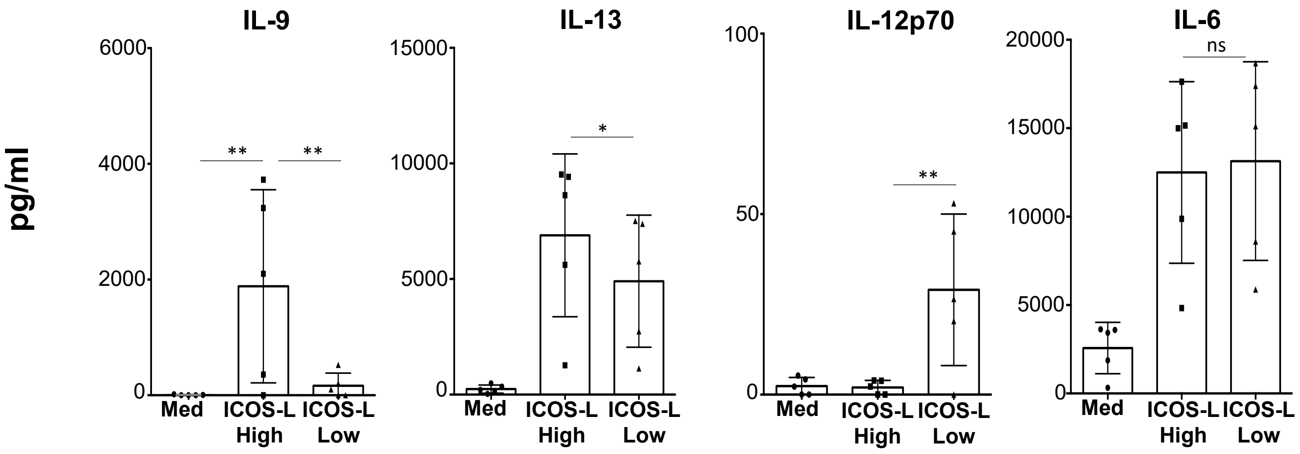

Fig. S5. Cytokine profile of CD4⁺ T cells induced by either ICOS-L^{High} or ICOS-L^{Low} GM-CSF-DC. (A) CBA (IL-9, IL-13, IL-12p70, and IL-6) assay for the measurement of cytokines in the supernatants of CD4$^+$ T cells differentiated either by ICOS-L^{High} or ICOS-L^{low} GM-CSF-activated DC, after additional 24h stimulation with aCD3/ aCD28 beads. Data are mean ± SEM from 5 independent experiments (n=5), *, $P < 0.05$; **, $P < 0.01$; ***, $P < 0.001$, by Wilcoxon or Student's test.

Journal of Cell Science • Supplementary information

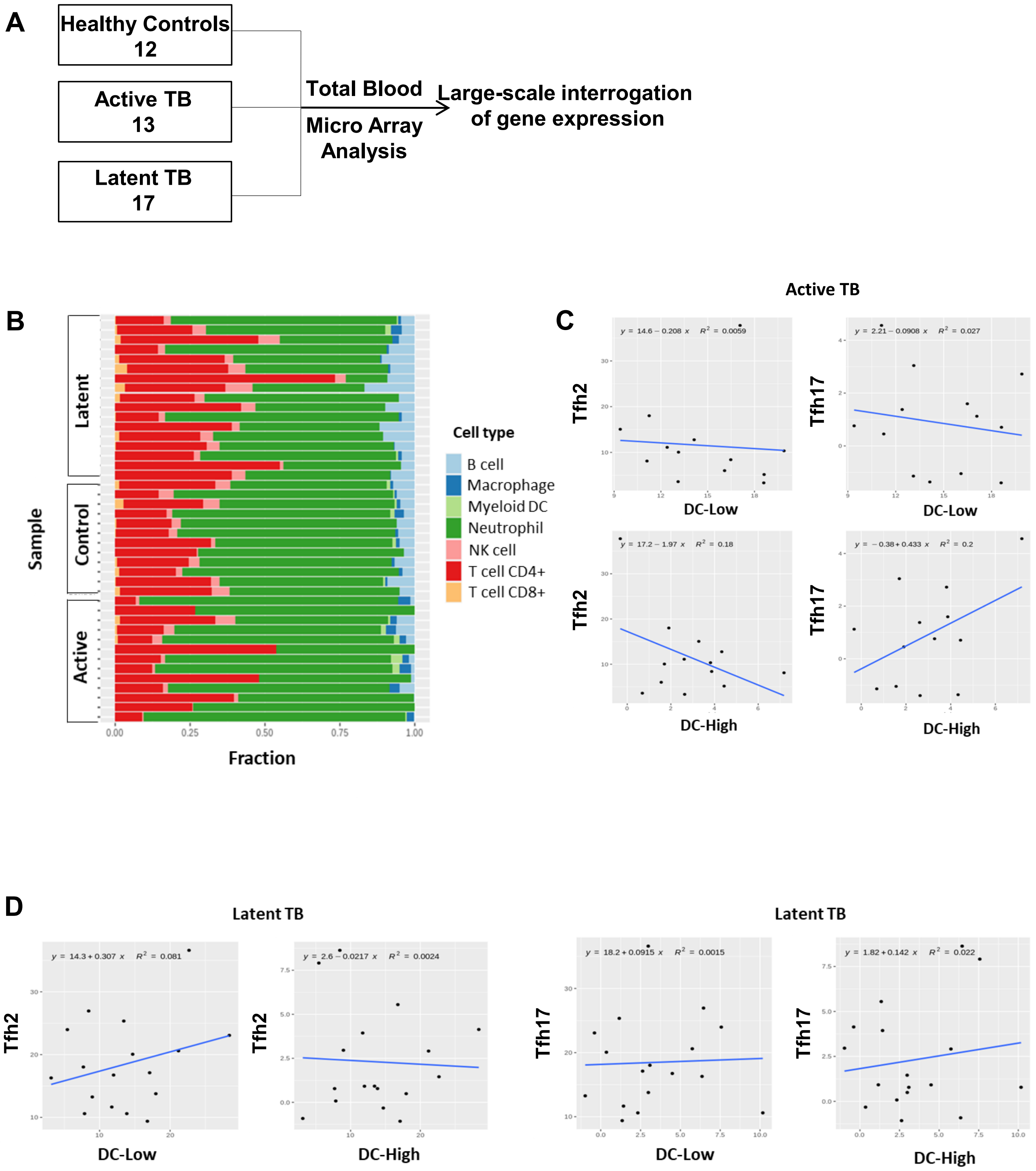

Fig. S6. Correlation of in-house constructed DC-High and DC-low signatures with Tfh1 or Tfh2 cells in MTB patients. (A) Experimental design of the training set by Berry et al (GSE19904) to decipher the transcriptional signatures of tuberculosis patients. Microarray analysis was performed on Whole Blood (WB) samples collected from healthy controls (n=12), active tuberculosis patients (n=13) and latent TB patients (n=17). (B) Deconvolution of the Berry London training set revealed the presence of the major immune cell types. The deconvolution was performed using Quantiseq method from the immunedeconv R package. This method outputs the fractions of each cell type within each sample and therefore, allows inter sample and inter cell type comparisons. (C) and (D) Collinearity assessment of the corresponding signatures of Tfh2 and Tfh17 with the respective DC subtype signatures DC-Low and DC-High within active and latent TB patients respectively. Each dot represents a sample. A linear regression model was applied to fit the dots repartition.

Journal of Cell Science • Supplementary information

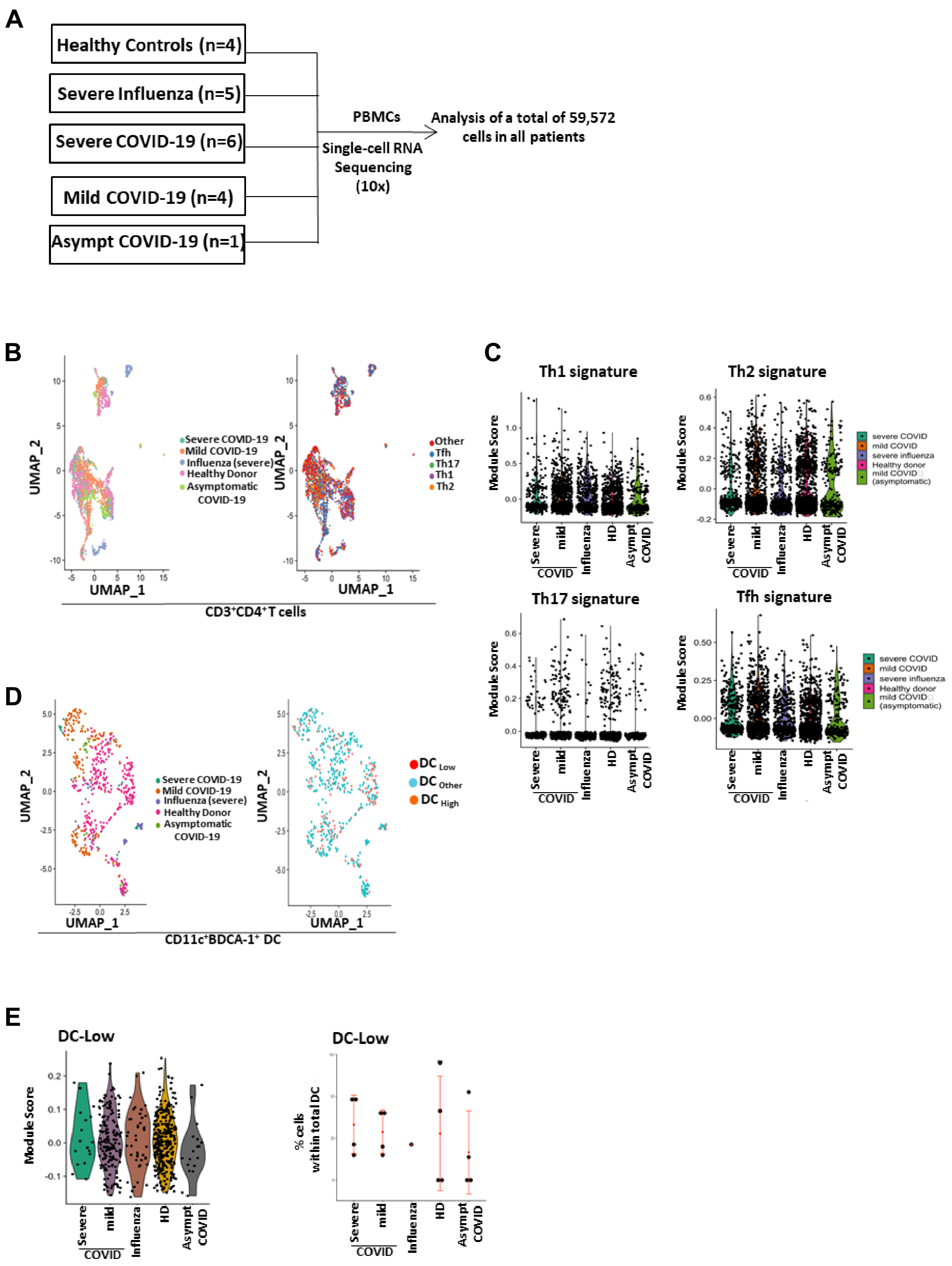

Fig. S7. Tfh1 cells are positively correlated with the presence of DC-Low signature in mild COVID-19 patients. (A) Experimental design to decipher correlation between DC and Tfh signatures in COVID-19 patients. scRNAseq analysis was performed in PBMC from healthy controls or active COVID-19 (mild and severe) and severe influenza patients. **(B)** UMAP representation of CD3$^+$CD4$^+$ T cells in all patient groups (left) as well as of different T-helper signatures (right). **(C)** Violin plot representation for the expression level of Th1-, Th2-, Th17- and Tfh-related signatures in all patient groups. **(D)** UMAP representation of CD11c$^+$BDCA1$^+$ DC in all patient groups and representation for the distribution of cells expressing signatures for ICOS-L^{Low} (DC-Low) and ICOS-L^{High} (DC-High) GM-CSF-activated DC based on the top 10 genes detected by the transcriptomic analysis. **(E)** Violin plot representation for the expression level of DC-Low signature in all disease groups as well as the percentages of cells positive for the DC-Low signature among the total DC detected in each patient group.

Table S1. Molecular signatures used for the identification of Tfh, Th1, Th2 and Th17 cells.
Tfh: T follicular helper cells; BCL6: B-cell lymphoma 6 protein; CXCR5: C-X-C chemokine receptor type 5; PDCD1: Programmed cell death 1 (PD-1); IL21: Interleukin-21; Th1: T helper 1 cells; TNF: Tumor necrosis factor; IFNG: Interferon Gamma; TBX21: T-box transcription factor (T-BET); Th2: T helper 2 cells; IL-4: Interleukin-4; IL-5: Interleukin-5; GATA3: GATA binding protein 3; Th17: T helper 17 cells; IL17A: Interleukin-17A; IL17F: Interleukin-17F; RORC: RAR related orphan receptor C.

**Tfh**	**Th1**	**Th2**	**Th17**
BCL6<br>CXCR5<br>PDCD1<br>IL21	TNF<br>IFNG<br>TBX21	IL4<br>IL5<br>GATA3	IL17A<br>IL17F<br>RORC

Table S2. Molecular signatures used for the identification of Tfh, Th1, Th2, Th17, Tfh1, Tfh2, and Tfh17 cells. Tfh: T follicular helper cells; ICOS: Inducible T Cell costimulator; PDCD1: Programmed cell death 1 (PD-1); CXCR5: C-X-C chemokine receptor type 5; IL21: Interleukin-21; BCL6: B-cell lymphoma 6 protein; Th1: T helper 1 cells; TNF: Tumor necrosis factor; IFNG: Interferon Gamma; TBX21: T-box transcription factor (T-BET); Th2: T helper 2 cells; IL-4: Interleukin-4; IL-5: Interleukin-5; GATA3: GATA binding protein 3; Th17: T helper 17 cells; IL17A: Interleukin-17A; IL17F: Interleukin-17F; RORC: RAR related orphan receptor C; Tfh1: type 1 T follicular helper cells; CXCR3: C-X-C Motif Chemokine Receptor 3; Tfh2: type 2 T follicular helper cells; Tfh17: type 17 T follicular helper cells; CCR6: C-C Motif Chemokine Receptor 6.

**Tfh**	**Th1**	**Th2**	**Th17**	**Tfh1**	**Tfh2**	**Tfh17**
ICOS<br>PDCD1<br>CXCR5<br>IL21<br>BCL6	TNF IFNG<br>TBX21	IL4<br>IL5<br>GATA3	IL17A<br>IL17F<br>RORC	ICOS<br>PDCD1<br>CXCR5<br>IL21<br>BCL6<br>TNF<br>TBX21<br>IFNG	IL4<br>IL5<br>GATA3<br>ICOS<br>PDCD1<br>CXCR5<br>IL21<br>BCL6	ICOS<br>PDCD1<br>CXCR5<br>IL21<br>BCL6<br>IL17A<br>IL17F
				CXCR3		CCR6

Journal of Cell Science • Supplementary information

Table S3. Top-9/10 differentially expressed genes characterizing the two in-vitro generated GM-CSF-activated DC subsets.

DC-High refers to top genes recovered from the transcriptomic analysis of the ICOS-L-High subset where and DC-Low refers to genes recovered from the ICOS-L-Low subset. Differential expression analysis was performed using DESeq2 R package. The genes were ranked according to their respective Fold Change (FC) values. DC-High signature is composed of genes with FC> 50, whereas the DC-Low signature includes genes with FC < -50.

**ICOS-L^{High} DC**<br><br>**"DC-High"**	**ICOS-L^{Low} DC**<br><br>**"DC-Low"**
ASXL3	CATSPER1
RNF17	BCAN
CLUHP3	CD302
SULF1	FAM81A
ADORA2A	ARHGEF15
HNF1B	TRIM10
MYH7	SNX29P1
SERPINE2	NME1-NME2
EDIL3	
