# Supplementary material for: GM-CSF-activated human dendritic cells promote type 1 T follicular helper cell polarization in a CD40-dependent manner
Source: J Cell Sci. 2022 Nov 11;135(21):jcs260298. doi: 10.1242/jcs.260298 (PMC9687542; doi:10.1242/jcs.260298)
Supplement: Supplementary file 1 [file joces-135-260298_review_history.pdf]

GM-CSF-activated human dendritic cells promote Tfh1 cell polarization in a CD40-dependent manner

Sarantis Korniotis, Melissa Saichi, Coline Trichot, Caroline Hoffmann, Elise Amblard, Annick Viguier, Sophie Grondin, Floriane Noel, Hamid Mattoo and Vassili Soumelis
DOI: 10.1242/jcs.260298

Editor: Daniel Billadeau

Review timeline
Submission to Review Commons: 10 Jan 2022
Original submission: 1 June 2022
Editorial decision: 9 June 2022
First revision received: 2 September 2022
Accepted: 5 October 2022

Reviewer 1

Evidence, reproducibility and clarity

Korniotis et al. identify that GM-CSF stimulates a subset of dendritic cells (DCs) that specifically promote Tfh1 cell differentiation. The authors set up a co-culture of DCs in media supplemented with either LPS, GM-CSF, or no supplements, and allogeneic human CD4+ T cells. scRNA-seq of the cultured CD4+ T cells demonstrated that only GM-CSF-DCs were able to promote a Tfh cell gene signature and specifically a Tfh1 cell profile. Further characterization of the Tfh cells from this co-culture revealed the GM-CSF-DCs promoted an increase in gene expression and protein secretion of TNFa, IL-21, IFNg, and CXCL13 in these CD4 T cells. Next, the authors showed that CD4 T cells activated by GM-CSF-DCs had increased surface expression of the canonical Tfh cell markers ICOS, PD-1 and CXCR5 relative to CD4 T cells activated by DCs from the other conditions. This Tfh cell phenotype was accompanied by an increase in the Tfh1 cell specific transcription factor expression of BCL6 and TBET, confirming their transcriptional data. The author's showed that cultured GM-CSF-DCs differentiate into two subsets defined by CD40 and ICOS-L expression. They found that the CD40hiICOS-Llo population of GM-CSF-DC is the primary driver of Tfh1-like cell differentiation. Further mechanistic studies of this DC-Tfh cell interaction showed that blocking CD40 was necessary for Tfh1 cell development while ICOS-L had minimal involvement.
The manuscript addresses and open question in the field, as the to which human DC subset promotes Tfh1 cell differentiation. The experiments are well thought out and controlled and they provide data to support their conclusions. However, there are aspects of Tfh cell differentiation and function that are not assessed by the experiments as discussed below.
The manuscript contains many grammatical mistakes and is at times hard to read. Overall, the authors show convincing evidence that GM-CSF-DCs promote Tfh1 cell differentiation in a CD40 dependent manner

Major Comments:
In figure 1 the authors show scRNA-seq data to identify the different effector CD4 T cell populations from their DC-T cell co-cultures. In figure 1A, the fourth panel they us UMAP to identify a "Tfh1 cells" and "pure Tfh cells", but it is unclear what genes signatures they are using to distinguish between these two populations. Furthermore, in Figure 1D the authors compare specific genes between Th2 and Tfh1 cells, but it is unclear how they are distinguishing Tfh1 and Th1 cells as TBET and IFNG are observed in both. They should also include CXCR3 as this is a gene associated with Tfh1 cells in mice (Sheikh et al Cell Reports 2019).

© 2022. Published by The Company of Biologists under the terms of the Creative Commons Attribution License (https://creativecommons.org/licenses/by/4.0/).

The authors address the functional development of the Tfh1 cells by co-culturing with naïve and memory B cells. The author use plasma cell differentiation of these cells to assess functional differences. However, they fail to assess an effector function of Tfh cells based on their cytokine production and that is class switch recombination. The authors should assess (primarily in naïve as memory may already be switched) secreted Ig from the supernatants to examine if the difference they observe in cytokine production (IFNg and TNFa) by these Tfh1 cells alters similar Ig switching. It has been shown that IL-21 alone promotes plasma cell differentiation in vitro.

The authors should address IL-12 and IL-2 receptor in their GM-CSF-DCs, two cytokines that have been shown to be essential by DCs in promoting the differentiation of Tfh cells (IL- 12-Schmitt et al Immunity 2009, Powell et al Sci. report 2019. IL-2R, Ditoro et al science 2018, Li et al Nature 2016)

Minor Comments:

All figures need to be more descriptive in their captions. Technique names and methodologies are often left out, leaving the reader to wonder how the experiment was set up. For example, legends do not include culture conditions, culture times, or quantification methods other than a statistical test (Figure 2A - "quantification after coculture" and axes are in pg/mL, so by what method?)

Many typos and grammatical mistakes such as:
In the abstract "Activated human blood CD1c+ DC" - should this not be "CD11c"?
- Page 5 in reference to figure 4 - there is a box after "GM-CSF receptor__chain", so I do not know what chain this is.
- Page 6 in reference to figure 5 - there is a typo about halfway down "phenotypic profile.cNext"
- Page 8 discussion - "transient TBET expression is sufficient to make Tfh cells secrete IFN- g for a long time", please define "long time"

Page 4 in reference to figure 3 - why not just call these "PD-1lo T cells"? "Tlow" is misleading since CXCR5 is still high.

Page 7 in reference to figure 8 - the DC-High and DC-Low nomenclature is misleading because it is as if there are high or low levels of DCs. I would advise sticking with ICOS- Lhigh/low DCs, particularly since that is how you referred to these subsets for the rest of the paper

Page 8 discussion - you pose the question of "whether cytokine production can be dissociated from transcription factors" because your Tfh1-like cells express gamma more dependent on ICOS-L than GATA3 or TBET. This is not really surprising as the ICOS-L:ICOS interactions between the Tfh and germinal center B cell or Tfh and DC can lead to cytokine secretion that can signal through the Tfh leading to a transcription factor aside from GATA3 and/or TBET.

Significance

The manuscript identifies a subset of GM-CSF derived DCs that promote human Tfh1 cell differentiation and highlight the role of CD40 in this process.

This manuscript fits into a growing literature about the mechanisms involved in human in vitro Tfh cell differentiation.

It would be applicable to a general immunology audience as these Tfh cells have a relevance to infections and autoimmunity.

I have an expertise in Tfh cell biology, adaptive immunity and humoral responses.

<u>**Reviewer 2**</u>

Evidence, reproducibility and clarity

See below.

© 2022. Published by The Company of Biologists under the terms of the Creative Commons Attribution License (https://creativecommons.org/licenses/by/4.0/).

Significance

In the present study, Soumelis et al. investigated that CD40 signaling from GM-CSF- activated dendritic cells is essential to promote Tfh1 cell polarization mainly using in vitro cell culture. Using multiplex omic methods the authors respectively analyzed different sequencing datasets including their own RNA-seq as well as the microarray and scRNA-seq data deposited by others to further investigate the relationship between DC subsets and Tfh- Tfh1 polarization. The authors concluded that by blocking CD40 signaling using antibody, Tfh1 polarization by GM-CSF activated dendritic cells was largely impaired, evidenced by the reduced frequency of IL-21+TNF-a+ Tfh-like cells in vitro. Overall, this is an interesting technical study. While the majority results of this study are from in vitro cell culture, which raises the significant concern on how much physiological relevance this GM-CSF-activated- DC potentiated Tfh1 polarization can provide. I appreciate the authors efforts analyzing human COVID-19 and MTB data to locate the correlation between such DC subsets and Tfh1 cells but lack of the in vivo investigation using animal models makes this study more suit for a method/technical paper to differentiate Tfh1-like cells in vitro for other experimental purpose.

Reviewer 3

Evidence, reproducibility and clarity

In this manuscript Korniotis et al reveal GM-CSF can activate a subset of DCs that are subsequently capable of promoting the generation of Tfh1 type cells. Some interesting data but a lot of it lacks appropriate controls with medium and LPS. In a lot of the figures, the FACS data is not convincing, and gates seem to be "randomly" determined. Dot plots rather than contour plots need to be used throughout the manuscript - it is clear some of the results are based on very few cells and therefore not convincing nor robust. Furthermore, there is a lack of functional data to determine if these Tfh1 cells generated by GMCSF DCs are B cell helpers (the true definition of a bona fide Tfh cell). The lack of proof reading, with numerous typographical errors and mislabeled graphs also puts doubt on the rigour of their results.

I reviewed this paper for another journal, and they have not changed a single word, including simple changes such as the typos and the mislabeling of axis I picked up! They didn't even bother to check the validity of the data where I pointed out inconsistencies between the text and the actual figure the data was presented! This is disappointing and frankly a waste of everyone's time as the authors didn't even attempt to alter the manuscript based on previous peer-reviews by multiple reviewers, before submission to another journal!

The following was raised in my previous review and still needs to be addressed:

Major comments:

- Dot plots rather than contour plots should be used throughout the manuscript - it is clear there are very few events/cells and this needs to be taken into consideration when considering the result - contour plots hide the number of cells present. For example, there may only be 100 cells present total and only 10 cells in the positive gate which would look like a real population in a contour plot.
- Fig1 - subsequent results reveal while the Th1 signature and Tfh signature overlap and represent 11.6%, there are clear IFNg only and IL-21 only cells (ie Fig2B). Why can't Th1 cells be differentiated as a separate signature?
- Fig2B - why are there no IL-4 expressing cells even though a clear Th2 signature is present (Fig 1A; 12%)?
- Page 4, last paragraph - PD1 co-expression with CXCR5 38.58% and ICOS 29.175% - are these data correct as data in Fig3B does not reflex these percentages.
- FigS2B - there are clear IL-9 secreting cells in response to GM-CSF DCs and IL-17A/IL-17- f in response to LPS DCs - why can't these signatures picked up in sc-RNAseq of co- cultured CD4+ T cells (Fig1A)?
- FigS2D - how are the gates determined? Why is IL-21 expression in Fig2C so different to 2D? Are they actually all IL-21 positive cells in D? similar for IFNg - not very convincing data when a lot of it is on the axis.

© 2022. Published by The Company of Biologists under the terms of the Creative Commons Attribution License (https://creativecommons.org/licenses/by/4.0/).

- Why is there so much TNF even after culture with medium ie in the absence of any simulation (Fig 2A, B)?
- Fig 3E - how were the gates for TBET and RORGT determined? Especially for TBET in the DN - there appears to be a positive and negative population similar to the Tlow and this appears to be ignored in the DN. Similar for RORGT in DN - is the higher of the 3 populations actually RORGT positive?
- FigS3 - result is not very convincing. DN population needs to be included as a control. Less than 0.1% of B cells are alive after co-culture with Tfh-like cells induced by GM-CSF DCs - how can one base results on 0.1% live B cells? Can Ig secretion be used as another readout? At present there are no convincing functional readouts of Tfh cell function (for GMCSF induced Tfh1 cells as well as ICOSlow and ICOShigh populations).
- Fig4 - why was LPS activated DC not included? Are these markers and populations a consequence of activation rather than GM-CSF? LPS control needs to be included to rule this out. Similarly, are ICOShigh and ICOSlow populations specifc to GMCSF DCs or are these populations present following activation with other stimuli such as LPS DCs?
- Fig5 - again it is unclear how the gates were determined. Medium and LPS controls need to be included. In particular, the background for IL-21 expression is extremely high and not very convincing. Why is the IL-21 staining in Fig5D so different to that of Fig 2B?
- Fig7A, B - I am surprised CD40 blocking had any affect on ICOShigh population as they don't; express high levels of CD40 (Fig 4C, D)
- Fig 7C - again why is the background for IL-21 staining so high compared to Fig 2B? the data is not very convincing. Similar comments for IL-4 expression - why is the background so high compared to Fig5D?
- Fig8, TB patients - a lot of emphasis was based on these data, which was the least convincing. The R-value for most were low ie Fig8B r = 0.44 - 0.5. Are most of these differences a reflection of differences in Tfh cells which the authors themselves point out in the discussion?
- Fig 8, COVID patients - extremely small sample size to be able to make any conclusions!
- Fig8C - Tfh1 cells look comparable to Tfh2 cells to me!
- Fig8D - One can argue Tfh1 cells appear to be more abundant in patients with severe COVID compared to these with mild COVID? Hard to tell with small sample size and such a large range.
- How do the authors reconcile the original description of Tfh1 cells being poor B cell helpers (Morita et al. Immunity 2011), which was confirmed in studies of patients with high frequencies of Tfh1 cells (Ma et al. J Allergy Clin Immunol 2015)? This needs to be discussed.
- Given the data presented, the contribution of CD40 is exaggerated and should be removed from the title and significance statements.

Minor comments:

- There are numerous typographical errors throughout the manuscript and thorough proof reading is highly advised.
- Fig 1A the Th2 signature is labelled as 12% in the figure but referred to as 22% in the text - which is it?
- Page 4, second paragraph - reference to FigS2A, B mislabeled with reference to Fig2A, B.
- Fig S7C - too small to interpret results/data
- Fig8B bottom right graph - x-axis should read "DC-High"

Significance

Without the changes mentioned above I can not comment on the significance as the data is not very convincing.

© 2022. Published by The Company of Biologists under the terms of the Creative Commons Attribution License (https://creativecommons.org/licenses/by/4.0/).

Author response to reviewers' comments

1. General Statements [optional]
This section is optional. Insert here any general statements you wish to make about the goal of the study or about the reviews.

2. Description of the planned revisions

Please see below the plan of our revision.

# Reviewer 1

1 Furthermore, in Figure 1D the authors compare specific genes between Th2 and Tfh1 cells, but it is unclear how they are distinguishing Tfh1 and Th1 cells as TBET and IFNG are observed in both. They should also include CXCR3 as this is a gene.

Reply: We apologize for not having explained in a clear way the molecular signatures (set of genes) we used for the identification of all T-helper cell lineages including Tfh and Th1 cells. We already provided a detailed table (Table 1, Supplementary File) describing the molecular signatures used in our scRNAseq data (Supplementary file). We will refer to that table in the Figure legend 1 and we will improve the way we explain that in our manuscript. Specifically, we will keep a table in the supplementary file highlighting the group of genes used as molecular signatures for all T helper cell lineages. The figure legend of both Figure 1 and Figure S1 will be modified accordingly.

In addition, we agree with the suggestion of the reviewer to include CXCR3 in our scRNAseq analysis. We will explore the expression level of CXCR3 in our scRNAseq dataset to investigate in which cluster of identified T helper cells we observe the highest score.

2 The authors should address IL-12 and IL-2 receptor in their GM-CSF-DCs, two cytokines that have been shown to be essential by DCs in promoting the differentiation of Tfh cells (IL-12-Schmitt et al Immunity 2009, Powell et al Sci. report 2019. IL-2R, Ditoro et al science 2018, Li et al Nature 2016).

Reply: We totally agree with the comment of the reviewer on the important role of the IL-12 and IL-2 cytokine receptors in the crosstalk between DC and T cells for promoting Tfh cell differentiation. However, our functional studies with blocking antibodies were designed to validate our own data-driven hypotheses. Our decision to move forward with blocking experiments for ICOS-L and CD40 was driven by our transcriptomic analysis which showed clear differential expression of these genes between the two subsets of GM-CSF-induced DCs (ICOS-L-High and ICOS-L-Low). Neither IL-12 nor IL-2R came out in our transcriptomic analysis. However, we acknowledge the strong biological relation between CD40 and IL-12 (Kelshall et al., Ann N Y Acad Sci, 1996, Macatonia et al., Journal of Immunol, 1995, Schulz et al., Journal of Immunol, 2000, Snijders et al., International Immunol, 1998) and we are willing to perform additional functional experiments by blocking IL-12 (in a similar way we already did for CD40 and ICOS-L). Regarding IL-2R, as you can see in our figure 4A-4B, we already present the expression levels of CD25 (IL- 2R) by GM-CSF-DC after 48h of stimulation. The expression of CD25 was like Medium-DCs, strongly suggesting that GM-CSF might not favor the up-regulation of this molecule which might not be involved in Tfh1 polarization.

3. Description of the revisions that have already been incorporated in the transferred manuscript
Please insert a point-by-point reply describing the revisions that were already carried out and included in the transferred manuscript. If no revisions have been carried out yet, please leave this section empty.

# Reviewer 1

Major Comments
1 In figure 1 the authors show scRNA-seq data to identify the different effector CD4 T cell populations from their DC-T cell co-cultures. In figure 1A, the fourth panel they us UMAP to identify a

© 2022. Published by The Company of Biologists under the terms of the Creative Commons Attribution License (https://creativecommons.org/licenses/by/4.0/).

"Tfh1 cells" and "pure Tfh cells", but it is unclear what genes signatures they are using to distinguish between these two populations.

Reply: We apologize for not having made clear in our manuscript the molecular signatures used for the identification of T helper cell lineages in the scRNAseq data. In the supplementary file, we provide a table (Table 1) with the detailed set of genes we used as ''molecular signature'' for each T helper cell population. The term "Tfh1" cells was used to define the ''cluster'' of cells which co-express both Th1 and Tfh signatures. As you can see in the **Figure 1A**, when we applied our in-house Tfh signature in the scRNAseq, we observed overlap with cells expressing the Th1 signature. So, we named this ''cluster'' Tfh1 cells.

Minor Comments

1 All figures need to be more descriptive in their captions. Technique names and methodologies are often left out, leaving the reader to wonder how the experiment was set up. For example, legends do not include culture conditions, culture times, or quantification methods other than a statistical test (Figure 2A - "quantification after coculture" and axes are in pg/mL, so by what method?)

Reply: We thank the reviewer for spending time to carefully highlight both technical and methodology-related limitations. We have now carefully revised our manuscript and we improved the description of both figure legends and methods.

2 In the abstract "Activated human blood CD1c+ DC" - should this not be "CD11c"?

Reply: CD1c (BDCA-1) is a canonical marker for the identification of type 2 conventional dendritic cells. We mentioned this marker (CD1c) in our abstract to make it clear that we worked exclusively with CD1c+ primary blood DC and not total CD11c+ DCs.

3 Page 5 in reference to figure 4 - there is a box after "GM-CSF receptor chain", so I do not know what chain this is.

Reply: The box was indeed a typo mistake that has been now removed. The chain of the GM- CSF-Receptor we used is a chain.

4 Page 6 in reference to figure 5 - there is a typo about halfway down "phenotypic profile.cNext"

Reply: Corrected

5 Page 8 discussion - "transient TBET expression is sufficient to make Tfh cells secrete IFN-g for a long time", please define "long time"

Reply: This comment is based on a paper published in the Journal of Experimental Medicine that we cite accordingly (Fang et al, 2018). The authors reported that IFN-g-expressing Tfh cells are absent in T-bet deficient mice, but present in mice with T-bet deletion at late stages of T cell differentiation. These findings suggest that a transient expression of T-bet during the first stages of T cell differentiation is enough to lead to the generation of IFN-g-producing Tfh cels. The time will be specified in our revised manuscript.

6 Page 4 in reference to figure 3 - why not just call these "PD-1lo T cells"? "Tlow" is misleading since CXCR5 is still high.

Reply: We thank the reviewer for the suggestion. We took it into consideration, and we added PD-1lo in a parenthesis.

7 Page 7 in reference to figure 8 - the DC-High and DC-Low nomenclature is misleading because it is as if there are high or low levels of DCs. I would advise sticking with ICOS-Lhigh/low DCs, particularly since that is how you referred to these subsets for the rest of the paper

© 2022. Published by The Company of Biologists under the terms of the Creative Commons Attribution License (https://creativecommons.org/licenses/by/4.0/).

Reply: We thank the reviewer for the suggestion. We decided to use the term DC-high and DC- low (and not the previously used ICOS-L-High and ICOS-L-Low) since we created in-house signatures including only the top 10 genes sorted from our transcriptomic analyses. We now explain that change in a clearer way, so we avoid any confusion.

8 Page 8 discussion - you pose the question of "whether cytokine production can be dissociated from transcription factors" because your Tfh1-like cells express gamma more dependent on ICOS-L than GATA3 or TBET. This is not really surprising as the ICOS-L:ICOS interactions between the Tfh and germinal center B cell or Tfh and DC can lead to cytokine secretion that can signal through the Tfh leading to a transcription factor aside from GATA3 and/or TBET.

Reply: We thank the reviewer for this comment. We added a new phrase in the discussion part explaining why we observe high expression levels of GATA-3 and/or RoRgT in IFN-g- expressing Tfh cells.

# Reviewer 2

1 Overall, this is an interesting technical study. While the majority results of this study are from in vitro cell culture, which raises the significant concern on how much physiological relevance this GM-CSF-activated-DC potentiated Tfh1 polarization can provide. I appreciate the authors efforts analyzing human COVID-19 and MTB data to locate the correlation between such DC subsets and Tfh1 cells but lack of the in vivo investigation using animal models makes this study more suit for a method/technical paper to differentiate Tfh1-like cells in vitro for other experimental purpose.

Reply: Physiology can very well be addressed in human systems, and human studies led to major discoveries without any use of animal models. One example is the discovery of the function of human TSLP and its link to allergy (Soumelis et al, Nat Immunol, 2002). Many more examples can be found following scRNAseq studies, which identified immune cell subsets, together with their molecular features (Nakamizo et al., J Exp Med, 2021, Dutertre et al., Immunity, 2019, See et al., Sience, 2019, Papalexi et al., Nat Rev Immunol, 2018, Villani et al., Science, 2017,) In our study, we use such scRNAseq approaches to identify Tfh1 subset, generate hypotheses. We identified novel mechanisms to generate Tf1 cells, and suggested physiopathological implications. As such, our study meets the highest possible standards for a human immunology study.

Reviewer 3

Major Comments

1 Dot plots rather than contour plots should be used throughout the manuscript - it is clear there are very few events/cells and this needs to be taken into consideration when considering the result - contour plots hide the number of cells present. For example, there may only be 100 cells present total and only 10 cells in the positive gate which would look like a real population in a contour plot.

Reply: We thank the reviewer for this suggestion to switch from a contour plot presentation of the Flow Cytometry analysis to dot plots. Our decision to present our data by contour plot was motivated by the specific recommendation of a high number of scientific journals, including Nature group, which support this type of presentation as the best way to highlight a larger amount of information such as cell density and cell contribution. We do not hide any ''issues'' related to cell numbers and would be happy to provide cell numbers for each gate in a supplementary table.

2 Fig1 - subsequent results reveal while the Th1 signature and Tfh signature overlap and represent 11.6%, there are clear IFNg only and IL-21 only cells (ie Fig2B). Why can't Th1 cells be differentiated as a separate signature?

Reply: We thank the reviewer for this interesting observation. Indeed, we were expecting even a small separate cluster of Th1 cells which would not overlap with Tfh-expressing cells. However, our scRNAseq dataset did not show well separated clusters which might be due to limitations of the experimental approach and/or the in-house molecular signatures we used for the identification of

© 2022. Published by The Company of Biologists under the terms of the Creative Commons Attribution License (https://creativecommons.org/licenses/by/4.0/).

Tfh cells. There is not a lot of available information for unique features of Tfh1 cells so we designed our set of genes based on a limited number of transcription factors and phenotypic markers which might not be sufficient to distinguish Th1 and Tfh cells in the scRNAseq data set. Therefore, we performed a big range of experiments in a protein level to better understand the complicated biology behind the human Tfh1 cell differentiation.

3 Fig2B - why are there no IL-4 expressing cells even though a clear Th2 signature is present (Fig 1A; 12%)?

Reply: The comment of the reviewer refers to the scRNAseq data. Indeed, in scRNAseq analysis we could confirm that there was a significant expression of IL-4 exclusively within the Th2 identified ‘’cluster’’. However, we could not detect significant levels of IL-4 at the protein level.

This might be due to additional biological factors which might inhibit cytokine secretion and detection in the time point we used (Induction of miRNas blocking the translation from mRNA into a protein). Moreover, when we analyzed T cells differentiated by each subset of GM-CSF-DC separately, we could detect increase of IL-4 induced by the ICOS-L-High subset, suggesting that a different biological environment enhanced the detection of the cytokine. To present our results in a clear way, we provided data not only through Flow Cytometry but also in the supernatants. This is a requirement when working with such complex biological systems.

4 Page 4, last paragraph - PD1 co-expression with CXCR5 38.58% and ICOS 29.175% - are these data correct as data in Fig3B does not reflex these percentages.

Reply: In Figure 3B, we provide a statistical analysis of percentages of both PD-1highCXCR5+ and ICOShighCXCR5+ cells based on pooled data as shown in Figure 3A. As can be seen in the related histograms, the average percentage of PD-1highCXCR5+ cells is between 30-40%, while the percentage of ICOShighCXC5+ cells is between 20-30%. Both average percentages reflect the average numbers we provide within the manuscript.

5 FigS2B - there are clear IL-9 secreting cells in response to GM-CSF DCs and IL-17A/IL-17-f in response to LPS DCs - why can't these signatures picked up in sc-RNAseq of co-cultured CD4+ T cells (Fig1A)?

Reply: This is a very common issue that has been raised many times in the field of scRNAseq. We know that one of the limitations of the scRNAseq method and especially, the 10X technology that we used in our study, is the sensitivity in identifying significant expression of several genes. For example, it is quite often that in scRNAseq analysis of activated T cells, the expression of PD- L1 cannot be detected. This is why in our study scRNAseq was used as hypothesis-generating, and we followed up with protein level validation of major data-derived hypotheses.

6 FigS2D - how are the gates determined? Why is IL-21 expression in Fig2C so different to 2D? Are they actually all IL-21 positive cells in D? similar for IFNg - not very convincing data when a lot of it is on the axis.

Reply: We thank the reviewer for this comment. This change might be due to several factors. Different lots of flow cytometry antibodies might be more brilliant than the previously used ones. Additionally, the second and biggest part of experiments was performed with the same type – but different – flow cytometry machine where the settings were different All the experiments using electronically sorted subsets of GM-CSF-DC were performed using a different machine with different settings. To avoid any confusion, we decided to add a phrase in the Material and Methods part for a better clarity.

7 Why is there so much TNF even after culture with medium ie in the absence of any simulation (Fig 2A, B)?

Reply: This is a very common feature of primary T cells. We have observed that many times over different projects, and it might be due to the presence of serum which might trigger the secretion of this easily produced cytokine. In our previously published paper (Pattarini et al., Journal of

© 2022. Published by The Company of Biologists under the terms of the Creative Commons Attribution License (https://creativecommons.org/licenses/by/4.0/).

Experimental Medicine, 2017), we obtained similar results about TNF-a.
8 Fig 3E - how were the gates for TBET and RORGT determined? Especially for TBET in the DN - there appears to be a positive and negative population similar to the Tlow and this appears to be ignored in the DN. Similar for RORGT in DN - is the higher of the 3 populations actually RORGT positive?
Reply: All our gating strategy was based on the use of isotype controls.

9 Fig4 - why was LPS activated DC not included? Are these markers and populations a consequence of activation rather than GM-CSF? LPS control needs to be included to rule this out. Similarly, are ICOShigh and ICOSlow populations specifc to GMCSF DCs or are these populations present following activation with other stimuli such as LPS DCs?

Reply: We thank the reviewer for this comment. We decided to focus exclusively on the maturation/activation profile of GM-CSF-DC since we have already reported in our previous figures that GM-CSF-DC is the only experimental condition (as compared to LPS-DC) able to induce significant Tfh1 cell differentiation. In addition, DC diversification was a unique feature of GM-CSF activation, and not seen with LPS-DC. Hence, we used only GM-CSF-DC to perform further experimental and functional validations.

10 Fig5 - again it is unclear how the gates were determined. Medium and LPS controls need to be included. In particular, the background for IL-21 expression is extremely high and not very convincing. Why is the IL-21 staining in Fig5D so different to that of Fig 2B?

Reply: Please, see our replies in the comments No 8 and No 9.

11 Fig7A, B - I am surprised CD40 blocking had any effect on ICOShigh population as they don't; express high levels of CD40 (Fig 4C, D)

Reply: The ICOS-L-High subset of GM-CSF-DCs expresses low levels of CD40. We were also surprised by the fact that inhibition of CD40 expression in low levels is sufficient to significantly modulate the Tfh identity. This suggests a previously undescribed role of CD40 in human Tfh cell differentiation.

12 Fig 7C - again why is the background for IL-21 staining so high compared to Fig 2B? the data is not very convincing. Similar comments for IL-4 expression - why is the background so high compared to Fig5D?

Reply: Please see our reply in the comment Number 8

13 **a)** Fig8, TB patients - a lot of emphasis was based on these data, which was the least convincing. The R-value for most were low ie Fig8B r = 0.44 - 0.5. Are most of these differences a reflection of differences in Tfh cells which the authors themselves point out in the discussion

b) Fig 8, COVID patients - extremely small sample size to be able to make any conclusions!

Reply: First, we tried to pick up the best data sets available in a literature-driven way since we wanted to use disease models where there is already evidence of the role of Tfh1 cells. So, these were the most convenient data sets we could find. Secondly, our conclusions are not only based on these findings, but in a large group of collective data. We provided data generated from a big range of experimental methods such as scRNAseq, transcriptomics, protein analysis and functional assays. All results from all the experiments support these conclusions. Indeed, if our conclusions were only based on our last figure, we would not be able to definitively support our findings. This last figure is just an additive piece in a puzzle of interesting and innovative findings we collected to demonstrate new evidence on human Tfh polarization. All these analyses suggest a physiopathological implication but do not constitute a definite conclusion.

14 Fig8C - Tfh1 cells look comparable to Tfh2 cells to me!

© 2022. Published by The Company of Biologists under the terms of the Creative Commons Attribution License (https://creativecommons.org/licenses/by/4.0/).

Reply: The aim of this figure is to demonstrate the presence of the different subsets of Tfh cells within the different disease groups of covid and influenza patients. We wanted to confirm that these cell populations are present so we can count on that observation for the next correlation study.

15 Fig8D - One can argue Tfh1 cells appear to be more abundant in patients with severe COVID compared to these with mild COVID? Hard to tell with small sample size and such a large range.
Reply: Please see our reply on the comment number 13.

16 How do the authors reconcile the original description of Tfh1 cells being poor B cell helpers (Morita et al. Immunity 2011), which was confirmed in studies of patients with high frequencies of Tfh1 cells (Ma et al. J Allergy Clin Immunol 2015)? This needs to be discussed.

Reply: We thank the reviewer for this comment. Indeed, the first paper which reported the presence of different Tfh subsets, demonstrated that Tfh1 cells might be poor B cell helpers. Since then, there is a big number of papers which identified Tfh1 cells in different disease models and showed a correlation between Tfh1 and B cell differentiation. We do not know exactly what the role of Tfh1 cells in B cell function is. It might induce a poor or different type of antibody secretion, switch recombination and cytokine secretion. This needs to be further investigated.

17 Given the data presented, the contribution of CD40 is exaggerated and should be removed from the title and significance statements

Reply: Our study highlights the role of CD40 in Tfh1 polarization, induced by a specific subset of GM-CSF-DC. According to our knowledge, it is the first study which demonstrates a mechanism for human Tfh1 differentiation, and we strongly believe that this message will be helpful for the Tfh scientific community. The fact that blocking of CD40 has an effect in both the phenotype and functional properties (cytokine profile) of GM-CSF-DC-induces Tfh1 cells makes it a very interesting candidate for the manipulation of these cells.

Minor Comments

18 There are numerous typographical errors throughout the manuscript and thorough proof reading is highly advised

Reply: We thank the reviewer for spending time to carefully revise our manuscript. We tried to improve its typographical quality taking into consideration all his/her comments.

19 Fig 1A the Th2 signature is labelled as 12% in the figure but referred to as 22% in the text - which is it?

Reply: Corrected

19 Page 4, second paragraph - reference to FigS2A, B mislabeled with reference to Fig2A, B.

Reply: Corrected

20 Fig S7C - too small to interpret results/data

Reply: Please see our reply in comment 13

21 Fig8B bottom right graph - x-axis should read "DC-High"

Reply: Corrected

Description of analyses that authors prefer not to carry out
Please include a point-by-point response explaining why some of the requested data or additional analyses might not be necessary or cannot be provided within the scope of a revision. This can be due to time or resource limitations or in case of disagreement about the necessity of such

© 2022. Published by The Company of Biologists under the terms of the Creative Commons Attribution License (https://creativecommons.org/licenses/by/4.0/).

additional data given the scope of the study. Please leave empty if not applicable.

Reviewer 1

1 The authors address the functional development of the Tfh1 cells by co-culturing with naïve and memory B cells. The author use plasma cell differentiation of these cells to assess functional differences. However, they fail to assess an effector function of Tfh cells based on their cytokine production and that is class switch recombination. The authors should assess (primarily in naïve as memory may already be switched) secreted Ig from the supernatants to examine if the difference they observe in cytokine production (IFNg and TNFa) by these Tfh1 cells alters similar Ig switching. It has been shown that IL-21 alone promotes plasma cell differentiation in vitro.

Reviewer 3

1 FigS3 - result is not very convincing. DN population needs to be included as a control. Less than 0.1% of B cells are alive after co-culture with Tfh-like cells induced by GM-CSF DCs - how can one base results on 0.1% live B cells? Can Ig secretion be used as another readout? At present there are no convincing functional readouts of Tfh cell function (for GMCSF induced Tfh1 cells as well as ICOSlow and ICOShigh populations).

Reply: We thank both reviewers for raising this comment. We totally acknowledge the importance of better understanding the role of different subsets of Tfh cells in B cell differentiation and function. The aim of this study is to provide new evidence on possible mechanisms used by human DCs to induce Tfh polarization. We tried to emphasize on this aspect, mostly in the crosstalk between DC and T cells. We believe that our data support in a robust and definitive manner the induction of a Tfh1 identity by GM-CSF-DC. The Tfh1 identity is established based on a large range of data, including scRNAseq analysis, expression of transcription factors and cytokines by several experimental approaches. To better convince about the induction of functional Tfh cells, we tried to demonstrate their ability to induce plasma cell differentiation. However, the scope of this paper is not to provide a full functional characterization of induced Tfh cells.

We agree with the comment of the reviewer regarding the few CD19+ recovered B cells we used for studying plasma cell differentiation. This kind of experiments is quite challenging. It is known that the best culture conditions might vary between T cells and B cells. We had to make a choice in which medium to use. We believe that the serum-free X-VIVO medium might favor T cell survival and proliferation leading to a limited space for B cells. This might be a reason why we were not able to recover more significant numbers of CD19+ B cells. However, we thought that it would be important to include these data which further support the induction of functional Tfh cells.

Original submission

First decision letter

MS ID#: JOCES/2022/260298

MS TITLE: GM-CSF-activated human dendritic cells promote type1 T follicular helper cells (Tfh1) polarization in a CD40-dependent manner

AUTHORS: Sarantis Korniotis, Melissa Saichi, Coline Trichot, Caroline Hoffmann, Elise Amblard, Annick Viguier, Sophie Grondin, Floriane Noel, Hamid Mattoo, and Vassili Soumelis
ARTICLE TYPE: Research Article

I have read the reviews from Review Commons and your response and plans for submission of a revised manuscript. I agree that an in vivo animal model is not needed as requested by reviewer 2. Please do focus on improving the quality of the manuscript as highlighted by both reviewers 1 and 3.

© 2022. Published by The Company of Biologists under the terms of the Creative Commons Attribution License (https://creativecommons.org/licenses/by/4.0/).

Please ensure that you clearly highlight all changes made in the revised manuscript. Please avoid using 'Tracked changes' in Word files as these are lost in PDF conversion.

I should be grateful if you would also provide a point-by-point response detailing how you have dealt with the points raised by the reviewers in the 'Response to Reviewers' box. Please attend to all of the reviewers' comments. If you do not agree with any of their criticisms or suggestions please explain clearly why this is so.

First revision

Author response to reviewers' comments

Point-by-point reply to Reviewer's comments

# Reviewer 1

1. In figure 1 the authors show scRNA-seq data to identify the different effector CD4 T cell populations from their DC-T cell co-cultures. In figure 1A, the fourth panel they us UMAP to identify a "Tfh1 cells" and "pure Tfh cells", but it is unclear what genes signatures they are using to distinguish between these two populations.

Reply: We apologize for not having made very clear the explanation of this part of the figure. We used four (4) different sets of gene signatures to identify the major T helper cell lineages: Tfh, Th1, Th2 and Th17 cells. The set of genes that we included in each group of T cells is described in a table that can be found in the supplementary file. Here is the table:

**Tfh**	**Th1**	**Th2**	**Th17**
BCL6<br>CXCR5<br>PDCD1<br>IL21	TNF<br>IFNG<br>TBX21	IL4<br>IL5<br>GATA3	IL17A<br>IL17F<br>RORC

Based on these molecular signatures, we could identify three well separated cell populations: Th1 - Th2 and Tfh cells. When we applied the molecular signature for Tfh cells, we could find two clusters. One cluster which was different from the other T-helper clusters that we named it as pure-Tfh cells. The second region where we had a high expression of the Tfh genes was overlapped with the region where we identified the Th1 cluster, therefore we named this region as Tfh1 cells.

2. Furthermore, in Figure 1D the authors compare specific genes between Th2 and Tfh1 cells, but it is unclear how they are distinguishing Tfh1 and Th1 cells as TBET and IFNG are observed in both. They should also include CXCR3 as this is a gene.

Reply: We apologize for not having explained in a clear way the molecular signatures (set of genes) we used for the identification of all T-helper cell lineages including Tfh and Th1 cells. We already provide a detailed table (Table 1, Supplementary File) describing the molecular signatures used in our scRNAseq data (Supplementary file). We now refer to that table in the Figure legend 1 and we improved the way we explain that in our manuscript.

In addition, we agree with the suggestion of the reviewer to include CXCR3 in our analysis.

To ensure the induction of Tfh1-like cells in our co-culture system of GM-CSF-DC and CD4+ T cells, at day 4 of co-culture, we performed additional staining of induced Tfh-like cells for the expression

© 2022. Published by The Company of Biologists under the terms of the Creative Commons Attribution License (https://creativecommons.org/licenses/by/4.0/).

of CCR6 and CXCR3. The differential expression of these two markers has been shown to identify three subsets of circulating Tfh cells. Tfh17 cells: CCR6+CXCR3-, Tfh2: CCR6-CXCR3+ and Tfh1 cells: CCR6- CXCR3+. As you can see in our figure below, more than 75% of Tfh-like cells induced by GM-CSF-DC are positive for the expression of CXCR3 and negative for the expression of CCR6 which fits well with the phenotypic identification of cTfh1 cells. Moreover, we could also identify a CCR6-CXCR3-population corresponding to cTfh2 cells. These data in a protein level, do confirm our scRNAseq analysis which revealed the polarization of naïve T cells mostly in Tfh1-like cells.

3. The authors address the functional development of the Tfh1 cells by co-culturing with naïve and memory B cells. The author use plasma cell differentiation of these cells to assess functional differences. However, they fail to assess an effector function of Tfh cells based on their cytokine production and that is class switch recombination. The authors should assess (primarily in naïve as memory may already be switched) secreted Ig from the supernatants to examine if the difference they observe in cytokine production (IFNg and TNFa) by these Tfh1 cells alters similar Ig switching. It has been shown that IL-21 alone promotes plasma cell differentiation in vitro.

Reply: We thank both reviewers for raising this comment. We totally acknowledge the importance of better understanding the role of different subsets of Tfh cells in B cell differentiation and function. The aim of this study is to provide new evidence on possible mechanisms used by human DC to induce Tfh polarization. We tried to emphasize on this aspect, mostly in the crosstalk between DC and T cells. We believe that our data support in a robust and definitive manner the induction of a Tfh1 identity by GM-CSF-DC. The Tfh1 identity is established based on a large range of data, including scRNAseq analysis, expression of transcription factors and cytokines by several experimental approaches. To better convince about the induction of functional Tfh cells, we tried to demonstrate their ability to induce plasma cell differentiation. However, the scope of this paper is not to provide a full functional characterization of induced Tfh cells.

4. The authors should address IL-12 and IL-2 receptor in their GM-CSF-DCs, two cytokines that have been shown to be essential by DCs in promoting the differentiation of Tfh cells (IL-12-Schmitt et al Immunity 2009, Powell et al Sci. report 2019. IL-2R, Ditoro et al science 2018, Li et al Nature 2016).

Reply: We totally agree with the comment of the reviewer in the important role of the cytokine IL-12 and IL-2 receptor in the crosstalk between DC and T cells for promoting Tfh cell differentiation. Our functional studies with blocking antibodies were mostly based on our data and not necessarily on previously published findings. Our decision to move forward with blocking experiments for ICOS-L and CD40 was driven by both literature research as well as our transcriptomic analysis which revealed clear differential expression of these genes between the two subsets of GM-CSF-induced DC (ICOS-L-High and ICOS-L-Low). Neither IL-12 nor IL-2R came out in our transcriptomic analysis. However, we acknowledge the strong biological relation between CD40 and IL-12 (Kelshall et al., Ann N Y Acad Sci, 1996, Macatonia et al., Journal of Immunol, 1995, Schulz et al., Journal of Immunol, 2000, Snijders et al., International Immunol, 1998) and we decided to perform additional functional experiments by blocking IL-12 (in a similar way we already did for CD40 and ICOS-L) in the co-culture systems using either total GM-CSF-DC or each subtype of GM-CSF-induced DC separately.

1. Co-culture system using total GM-CSF-DC:

As you can see in the figure below, blocking of IL-12 on GM-CSF-DC induced in T cells a cytokine profile quite like what we get when blocking CD40. It significantly reduced the frequencies of cells producing IL-21 and TNF-α (characterizing Tfh cells) and within this population of IL-21+TNF-α+ cells, we observed increase in the frequencies of IL-4-producing cells associated with a decrease in the frequencies of IFN-γ-producing cells. These results do highlight a possible link between CD40 and IL- 12 but does not exclude the possibility that other CD40-related mechanisms could be involved. Based on the already accepted relation between CD40 and IL-12, our findings do confirm the important role of CD40 in controlling Tfh1 polarization as displayed by human GM-CSF-induced DC.

2. Co-culture system using either ICOS-L-Low or ICOS-L-High GM-CSF-DC:

Since our study demonstrates that GM-CSF induces the emergence of two functionally different

© 2022. Published by The Company of Biologists under the terms of the Creative Commons Attribution License (https://creativecommons.org/licenses/by/4.0/).

subtypes of DC, we decided to test whether blocking of IL-12, in each subtype separately, does affect the subsequent cytokine profile of T cells. As you can see in the part (**c**) and (**d**) of the figure below, IL- 12 blocking is more active and potent in modulating the cytokine profile of T cells induced by ICOS-L- Low DC. In this condition, T cells displayed decreased frequencies of total IL-21+TNF-α+ cells, associated with an increase in the frequencies of IL-4-producing Tfh cells and decrease in IFN-γ- producing Tfh cells. On the other hand, the activity of IL-12 blocking in T cells induced by ICOS-L-High DC is much slighter since the cytokine profile is very similar to No blocking and CD40 condition.

Overall, these findings confirm that the ICOS-L-Low GM-CSF-DC is the dominant subset in inducing IFN-γ-producing Tfh cells and both secretion of IL-21 and IFN-γ seem to be affected by CD40 and IL-12. The CD40/IL-12 axis seems to be involved in human Tfh1 polarization. For the moment, we prefer to keep these newly generated data out of the current version of our paper but if the reviewer thinks that it is essential to improve the impact of our study, we will be glad to find place and include them.

Regarding IL-2R, as you can see in our figure 4A-4B, we already present the expression levels of CD25 (IL-2R) by GM-CSF-DC after 48h of stimulation. Apparently, the expression of CD25 is not much higher than Medium-DC, suggesting that GM-CSF might not favor the up-regulation of this molecule which might not be involved in Tfh1 polarization. Based on these preliminary data we already have, together with the belief that additional functional blocking experiments against other genes of interest will complicate the message of our study, we prefer to stay in a more CD40-related axis.

5 All figures need to be more descriptive in their captions. Technique names and methodologies are often left out, leaving the reader to wonder how the experiment was set up. For example, legends do not include culture conditions, culture times, or quantification methods other than a statistical test (Figure 2A - "quantification after coculture" and axes are in pg/mL, so by what method?)

Reply: We thank the reviewer for spending time to carefully highlight both technique - and methodology-related limitations. We have now carefully revised our manuscript and we improved the description of both figure legends and methods. However, due to word limitation in the main text, for the technical details, we prefer to keep all the information exclusively in the section of Materials and Methods and only mentioned in the figure legends when the experimental conditions change.

6 In the abstract "Activated human blood CD1c+ DC" - should this not be "CD11c"?

Reply: CD1c (BDCA-1) is a canonical marker for the identification of type 2 conventional dendritic cells. We used this marker (CD1c) in our abstract to make it clear that we worked exclusively with CD1c+ primary blood DC and not total CD11c+ DCs.

7 Page 5 in reference to figure 4 - there is a box after "GM-CSF receptor chain", so I do not know what chain this is.

Reply: The box was indeed a typo mistake that has been now removed. The chain of the GM-CSF-Receptor we used is a chain.

8 Page 6 in reference to figure 5 - there is a typo about halfway down "phenotypic profile.cNext"

Reply: Corrected

9 Page 8 discussion - "transient TBET expression is sufficient to make Tfh cells secrete IFN-g for a long time", please define "long time"

Reply: This comment is based on a paper published in Journal of Experimental Medicine that we cite accordingly (Fang et al, 2018). The authors reported that IFN-g-expressing Tfh cells are absent in T-bet deficient mice, but fully present in mice with T-bet deletion at late stages of T cell differentiation. These findings suggest that a transient expression of T-bet during only the first stages of T-cell differentiation is enough to lead to the generation of IFN-g-producing Tfh cels.

© 2022. Published by The Company of Biologists under the terms of the Creative Commons Attribution License (https://creativecommons.org/licenses/by/4.0/).

10 Page 4 in reference to figure 3 - why not just call these "PD-1lo T cells"? "Tlow" is misleading since CXCR5 is still high.

Reply: We thank the reviewer for the suggestion. We took it into consideration, and we added PD-1lo in a parenthesis.

11 Page 7 in reference to figure 8 - the DC-High and DC-Low nomenclature is misleading because it is as if there are high or low levels of DCs. I would advise sticking with ICOS-Lhigh/low DCs, particularly since that is how you referred to these subsets for the rest of the paper

Reply: We thank the reviewer for the suggestion. We decided to use the term DC-high and DC-low (and not the previously used ICOS-L-High and ICOS-L-Low) since we created in-house signatures including only the top 10 genes sorted from our transcriptomic analyses. We explained that change in a clear way in the Legend for Table 3, so we avoid any confusion.

12 Page 8 discussion - you pose the question of "whether cytokine production can be dissociated from transcription factors" because your Tfh1-like cells express gamma more dependent on ICOS-L than GATA3 or TBET. This is not really surprising as the ICOS-L: ICOS interactions between the Tfh and germinal center B cell or Tfh and DC can lead to cytokine secretion that can signal through the Tfh leading to a transcription factor aside from GATA3 and/or TBET.

Reply: We thank the reviewer for this comment. We added a new phrase in the discussion part explaining why we observe high expression levels of GATA-3 and/or RoRgT in IFN-g-expressing Tfh cells.

Reviewer 2

Overall, this is an interesting technical study. While the majority results of this study are from in vitro cell culture, which raises the significant concern on how much physiological relevance this GM-CSF- activated-DC potentiated Tfh1 polarization can provide. I appreciate the authors efforts analyzing human COVID-19 and MTB data to locate the correlation between such DC subsets and Tfh1 cells but lack of the in vivo investigation using animal models makes this study more suit for a method/technical paper to differentiate Tfh1-like cells in vitro for other experimental purpose.

Reply: Physiology can very well be addressed in human systems, and human studies led to major discoveries without any use of animal models. One example is the discovery of the function of human TSLP and its link to allergy (Soumelis et al, Nat Immunol, 2002). Many more examples can be found following scRNAseq studies, which identified immune cell subsets, together with their molecular features (Nakamizo et al., J Exp Med, 2021, Dutertre et al., Immunity, 2019, See et al., Sience, 2019, Papalexi et al., Nat Rev Immunol, 2018, Villani et al., Science, 2017). In our study, we use such scRNAseq approaches to identify Tfh1 subset and generate hypotheses. We identified novel mechanisms to generate Tf1 cells, and suggested physiopathological implications. As such, our study meets the highest possible standards for a human immunology study.

Reviewer 3

1 Dot plots rather than contour plots should be used throughout the manuscript - it is clear there are very few events/cells and this needs to be taken into consideration when considering the result - contour plots hide the number of cells present. For example, there may only be 100 cells present total and only 10 cells in the positive gate which would look like a real population in a contour plot.

Reply: We thank the reviewer for the suggestion to switch from a contour plot presentation of the Flow Cytometry analysis to dot plots. Our decision to present our data by contour plot was motivated by the specific recommendation of a high number of scientific journals, including Nature group, which support this type of presentation as the best way to highlight a bigger amount of information such as cell density and cell contribution. We do not hide a y ‘’ ssues’’ re ated t e um ers f the rev ewer wants, we can modify all our FACS presentations which will require a significant amount of time. Alternatively, we can provide some few examples in both presentations, so we

© 2022. Published by The Company of Biologists under the terms of the Creative Commons Attribution License (https://creativecommons.org/licenses/by/4.0/).

convince the reviewer about his/her concern.

2 Fig1 - subsequent results reveal while the Th1 signature and Tfh signature overlap and represent 11.6%, there are clear IFNg only and IL-21 only cells (ie Fig2B). Why can't Th1 cells be differentiated as a separate signature?

Reply: We thank the reviewer for this interesting observation. Indeed, we were expecting even a small separate cluster of Th1 cells which will not overlap with Tfh-expressing cells. Unfortunately, our scRNAseq analysis did not reveal well separated clusters which might be due to limitations of the experimental approach and/or the in-house molecular signatures we used for the identification of Tfh cells. There is not a lot of available information for unique features of Tfh1 cells so we designed our set of genes based on a limited number of transcription factors and phenotypic markers which might not be sufficient to distinguish Th1 and Tfh cells in the scRNAseq data set. Therefore, we performed a big range of experiments in a protein level to better understand the complicated biology behind the human Tfh1 cell differentiation.

3 Fig2B - why are there no IL-4 expressing cells even though a clear Th2 signature is present (Fig 1A; 12%)?

Reply: The comment of the reviewer refers to the scRNAseq data. Indeed, in scRNAseq analysis we could confirm that there was a significant expression of IL-4 exclusively within the Th2 identified ‘’ uster’’ H wever we u d t dete t s f a t evels of IL-4 in a protein level. This might be due to additional biological factors which create a kind of competition in the cytokine secretion and detection. The presence of other cytokines might inhibit the significant secretion of IL-4 by T cells in the case of total GM-CSF-DC. However, when we analyzed T cells differentiated by each subset of GM- CSF-DC separately, we could detect increase of IL-4 induced by the ICOS-L-High subset, suggesting that a different biological environment enhanced the detection of the cytokine. To present our results in a clear way, we provided data not only through Flow Cytometry but also in the supernatants. This is a requirement when working with such complex biological systems.

4 Page 4, last paragraph - PD1 co-expression with CXCR5 38.58% and ICOS 29.175% - are these data correct as data in Fig3B does not reflex these percentages.

Reply: In Figure 3B, we provide a statistical analysis of percentages of both PD-1highCXCR5+ and ICOShighCXCR5+ cells based on pooled data as shown in Figure 3A. As you can see in the related histograms, the average percentage of PD-1highCXCR5+ cells, is between 30-40% where the percentage of ICOShighCXC5+ cells, is between 20-30%. Both average percentages reflect the average numbers we provide within the manuscript.

5 FigS2B - there are clear IL-9 secreting cells in response to GM-CSF DCs and IL-17A/IL-17-f in response to LPS DCs - why can't these signatures picked up in sc-RNAseq of co-cultured CD4+ T cells (Fig1A)?

Reply: This is a very common issue that has been raised so many times in the field of scRNAseq. We know that one of the limitations of the scRNAseq method, and especially the 10X technology that we used in our study, is the sensitivity in identifying significant expression of several genes. For example, it is quite often that in scRNAseq analysis of activated T cells, the expression of PD- a ’t e detected. In this case, we strongly believe that the levels of protein secretion are more reliable and definitive.

6 FigS2D - how are the gates determined? Why is IL-21 expression in Fig2C so different to 2D? Are they actually all IL-21 positive cells in D? similar for IFNg - not very convincing data when a lot of it is on the axis.

Reply: We thank the reviewer for this comment. This change might be due to several factors. Different lots of flow cytometry antibodies might be more brilliant than the previously used ones. Additionally, the second and biggest part of experiments was performed with the same type – but different – Flow cytometry machine where the settings were different. To avoid any confusion, we decided to add a phrase in the Material and Methods part for a better clarity (say where exactly in

© 2022. Published by The Company of Biologists under the terms of the Creative Commons Attribution License (https://creativecommons.org/licenses/by/4.0/).

the manuscript).

7 Why is there so much TNF even after culture with medium ie in the absence of any simulation (Fig 2A, B)?

Reply: This is a very common feature of primary T cells. We have observed that many times during different projects and it might be due to the presence of serum which might trigger the secretion of this easily produced cytokine. You can see that in our previously published paper (Pattarini et al., Journal of Experimental Medicine, 2017), we had the same results about TNF-a. We do not think that it is a serious issue since the interpretation of the results is based always in a comparative manner.

8 Fig 3E - how were the gates for TBET and RORGT determined? Especially for TBET in the DN - there appears to be a positive and negative population similar to the Tlow and this appears to be ignored in the DN. Similar for RORGT in DN - is the higher of the 3 populations actually RORGT positive?

Reply: All our gating strategy was based on the use of isotype controls.

9 Fig4 - why was LPS activated DC not included? Are these markers and populations a consequence of activation rather than GM-CSF? LPS control needs to be included to rule this out. Similarly, are ICOShigh and ICOSlow populations specifc to GMCSF DCs or are these populations present following activation with other stimuli such as LPS DCs?

Reply: We thank the reviewer for this comment. We decided to focus exclusively on the maturation/activation profile of GM-CSF-DC since we have already reported in our previous figures that GM-CSF-DC is the only experimental condition (as compared to LPS-DC) able to induce significant
Tfh1 cell differentiation. Due to space limitations, we thought that it will be out of the scope of the paper to include the activation profile of LPS-DC. If the reviewer believes that the presentation of these results will augment the impact of our study, we will be more than happy to find a way to include them. However, we would like to mention that the DC diversification is a unique feature of GM-CSF activation.

10 Fig5 - again it is unclear how the gates were determined. Medium and LPS controls need to be included. In particular, the background for IL-21 expression is extremely high and not very convincing. Why is the IL-21 staining in Fig5D so different to that of Fig 2B?

Reply: Please see our replies in the comments No 8 and No 9.

11 Fig7A, B - I am surprised CD40 blocking had any affect on ICOShigh population as they don't; express high levels of CD40 (Fig 4C, D)

Reply: The ICOS-L-High subset of GM-CSF-DCs expresses low levels of CD40. We were also surprised by the fact that inhibition of CD40 expression in low levels is enough to significantly modulate the Tfh identity. This suggests a previously undescribed role of CD40 in human Tfh cell differentiation.

12 Fig 7C - again why is the background for IL-21 staining so high compared to Fig 2B? the data is not very convincing. Similar comments for IL-4 expression - why is the background so high compared to Fig5D?

Reply: Please see our reply in the comment Number 8

13 **a)** Fig8, TB patients - a lot of emphasis was based on these data, which was the least convincing. The R-value for most were low ie Fig8B r = 0.44 - 0.5. Are most of these differences a reflection of differences in Tfh cells which the authors themselves point out in the discussion, **b**) Fig 8, COVID patients - extremely small sample size to be able to make any conclusions!

Reply: We know that there are weaknesses in the data sets we used to demonstrate a possible physiological relevance of our in-vitro generated findings (sample size). First, we tried to pick up

© 2022. Published by The Company of Biologists under the terms of the Creative Commons Attribution License (https://creativecommons.org/licenses/by/4.0/).

the best data sets available in a literature-driven way since we wanted to use disease models where there is already evidence for the role of Tfh1 cells. So, these were the best data sets we could find. Secondly, our conclusions are not only based on these findings, but in a large group of collective data. We provided data generated from a big range of experimental methods such as scRNAseq, transcriptomics, protein analysis and functional assays. All results from all the experiments support these conclusions. Indeed, if our conclusions were only based on our last figure, we would not be able to definitively support our findings. This last figure is just an additive piece in a puzzle of interesting and innovative findings we collected to demonstrate new evidence on human Tfh polarization.

14 Fig8C - Tfh1 cells look comparable to Tfh2 cells to me!

Reply: The aim of this figure is to demonstrate the presence of the different subsets of Tfh cells within the different disease groups of covid and influenza patients. We wanted to confirm that these cell populations are present so we can count on that observation for the next correlation study.

15 Fig8D - One can argue Tfh1 cells appear to be more abundant in patients with severe COVID compared to these with mild COVID? Hard to tell with small sample size and such a large range.
Reply: Please see our reply on the comment number 13.

16 How do the authors reconcile the original description of Tfh1 cells being poor B cell helpers (Morita et al. Immunity 2011), which was confirmed in studies of patients with high frequencies of Tfh1 cells (Ma et al. J Allergy Clin Immunol 2015)? This needs to be discussed.

Reply: We thank the reviewer for this comment. Indeed, the first paper which reported the presence of different Tfh subsets, demonstrated that Tfh1 cells might be poor B cell helpers. Since then, there is a big number of papers which identified Tfh1 cells in different disease models and showed a correlation between tfh1 and B cell differentiation. We don't know exactly what the role of Tfh1 cells in B cell function is. It might induce a poor or different type of antibody secretion, switch recombination and cytokine secretion. This needs to be further investigated.

17 Given the data presented, the contribution of CD40 is exaggerated and should be removed from the title and significance statements

Reply: Our study highlights the role of CD40 in Tfh1 polarization, induced by a specific subset of GM- CSF-DC. According to our knowledge, it is the first study which demonstrates a mechanism for human Tfh1 differentiation, and we strongly believe that this message will be helpful for the Tfh scientific community.

18 There are numerous typographical errors throughout the manuscript and thorough proof reading is highly advised

Reply: We thank the reviewer for spending time to carefully revise our manuscript. We tried to improve its typographical quality taking into consideration all his/her comments.

19 Fig 1A the Th2 signature is labelled as 12% in the figure but referred to as 22% in the text - which is it?

Reply: Corrected

20 Page 4, second paragraph - reference to FigS2A, B mislabeled with reference to Fig2A, B.

Reply: Corrected

20 Fig S7C - too small to interpret results/data

Reply: Please see our reply in comment 13

21 Fig8B bottom right graph - x-axis should read "DC-High"

© 2022. Published by The Company of Biologists under the terms of the Creative Commons Attribution License (https://creativecommons.org/licenses/by/4.0/).

Reply: Corrected

22 FigS3 - result is not very convincing. DN population needs to be included as a control. Less than 0.1% of B cells are alive after co-culture with Tfh-like cells induced by GM-CSF DCs - how can one base results on 0.1% live B cells? Can Ig secretion be used as another readout? At present there are no convincing functional readouts of Tfh cell function (for GMCSF induced Tfh1 cells as well as ICOSlow and ICOShigh populations).

Reply: We thank both reviewers for raising this comment. We totally acknowledge the importance of better understanding the role of different subsets of Tfh cells in B cell differentiation and function. The aim of this study is to provide new evidence on possible mechanisms used by human DCs to induce Tfh polarization. We tried to emphasize on this aspect, mostly in the crosstalk between DC and T cells. We believe that our data support in a robust and definitive manner the induction of a Tfh1 identity by GM-CSF-DC. The Tfh1 identity is established based on a large range of data, including scRNAseq analysis, expression of transcription factors and cytokines by several experimental approaches. To better convince about the induction of functional Tfh cells, we tried to demonstrate their ability to induce plasma cell differentiation. However, the scope of this paper is not to provide a full functional characterization of induced Tfh cells.

We agree with the comment of the reviewer regarding the few CD19+ recovered B cells we used for studying plasma cell differentiation. This kind of experiments is quite challenging. It is known that the best culture conditions might vary between T cells and B cells. We had to make a choice in which medium to use. We believe that the serum-free X-VIVO medium might favor T cell survival and proliferation leading to a limited space for B cells. This might be a reason why we were not able to recover more significant numbers of CD19+ B cells. However, we thought that it would be important to include these data which further support the induction of functional Tfh cells.

Second decision letter

MS ID#: JOCES/2022/260298

MS TITLE: GM-CSF-activated human dendritic cells promote type1 T follicular helper cells (Tfh1) polarization in a CD40-dependent manner

AUTHORS: Sarantis Korniotis, Melissa Saichi, Coline Trichot, Caroline Hoffmann, Elise Amblard, Annick Viguier, Sophie Grondin, Floriane Noel, Hamid Mattoo, and Vassili Soumelis
ARTICLE TYPE: Research Article

Thank you for sending your manuscript to Journal of Cell Science through Review Commons.

I am happy to tell you that your manuscript has been accepted for publication in Journal of Cell Science, pending standard ethics checks.

Reviewer 1

Advance summary and potential significance to field

In the present study, Soumelis et al. investigated that CD40 signaling from GM-CSF-activated dendritic cells is essential to promote Tfh1 cell polarization mainly using in vitro cell culture. Using multiplex omic methods the authors respectively analyzed different sequencing datasets including their own RNA-seq as well as the microarray and scRNA-seq data deposited by others to further investigate the relationship between DC subsets and Tfh-Tfh1 polarization. The authors concluded that by blocking CD40 signaling using antibody Tfh1 polarization by GM-CSF activated dendritic cells was largely impaired, evidenced by the reduced frequency of IL-21+TNF-a+ Tfh-like cells in vitro.

© 2022. Published by The Company of Biologists under the terms of the Creative Commons Attribution License (https://creativecommons.org/licenses/by/4.0/).

Comments for the author

I appreciate the author's efforts to revise this manuscript and respond to my comments. I do not have further

concerns.

Reviewer 2

Advance summary and potential significance to field

This paper addresses identifying which human DC subset promotes Tfh1 cell differentiation. While a majority of their findings are found in vitro and may not completely translate in vivo. However, this study does suggest a new role for GM-CSF-DCs in Tfh cell differentiation.

Comments for the author

The authors made appropriate revisions and I have no further comments

© 2022. Published by The Company of Biologists under the terms of the Creative Commons Attribution License (https://creativecommons.org/licenses/by/4.0/).
